# Relative cerebral flow from dynamic PIB scans as an alternative for FDG scans in Alzheimer’s disease PET studies

**DOI:** 10.1371/journal.pone.0211000

**Published:** 2019-01-17

**Authors:** Débora E. Peretti, David Vállez García, Fransje E. Reesink, Tim van der Goot, Peter P. De Deyn, Bauke M. de Jong, Rudi A. J. O. Dierckx, Ronald Boellaard

**Affiliations:** 1 Department of Nuclear Medicine and Molecular Imaging, University Medical Center Groningen, University of Groningen, Groningen, Groningen, The Netherlands; 2 Department of Neurology, Alzheimer Research Centre, University Medical Center Groningen, University of Groningen, Groningen, Groningen, The Netherlands; 3 Institute Born-Bunge, Laboratory of Neurochemistry and Behaviour, University of Antwerp, Antwerp, Antwerp, Belgium; Nathan S Kline Institute, UNITED STATES

## Abstract

In Alzheimer’s Disease (AD) dual-tracer positron emission tomography (PET) studies with 2-[^18^F]-fluoro-2-deoxy-D-glucose (FDG) and ^11^C-labelled Pittsburgh Compound B (PIB) are used to assess metabolism and cerebral amyloid-β deposition, respectively. Regional cerebral metabolism and blood flow (rCBF) are closely coupled, both providing an index for neuronal function. The present study compared PIB-derived rCBF, estimated by the ratio of tracer influx in target regions relative to reference region (*R*_1_) and early-stage PIB uptake (ePIB), to FDG scans. Fifteen PIB positive (+) patients and fifteen PIB negative (-) subjects underwent both FDG and PIB PET scans to assess the use of *R*_1_ and ePIB as a surrogate for FDG. First, subjects were classified based on visual inspection of the PIB PET images. Then, discriminative performance (PIB+ versus PIB-) of rCBF methods were compared to normalized regional FDG uptake. Strong positive correlations were found between analyses, suggesting that PIB-derived rCBF provides information that is closely related to what can be seen on FDG scans. Yet group related differences between method’s distributions were seen as well. Also, a better correlation with FDG was found for *R*_1_ than for ePIB. Further studies are needed to validate the use of *R*_1_ as an alternative for FDG studies in clinical applications.

## Introduction

Several clinical studies were performed over the last years to validate biomarkers for evaluating the pathological mechanisms of Alzheimer's disease (AD). It is important to consider that different biomarkers may point at abnormality at particular stages of disease progression [1], thus providing varied information in the discernment between conditions with concomitant AD pathology, such as dementia with Lewy bodies [2], Parkinson's disease with dementia [3], and frontotemporal lobar degeneration [4].

Two of the main AD biomarkers are those related with the amyloid-β (Aβ) plaques deposition in the brain, a histopathology hallmark of the AD that has an important role in disease progression [1], and neurodegeneration biomarkers, which reflect synaptic dysfunction and degeneration. Moreover, the prodromal phase of AD, known as mild cognitive impairment (MCI), which is characterized by the onset of the earliest cognitive symptoms that do not meet dementia criteria, can present abnormal levels of these biomarkers [1].

In this context, positron emission tomography (PET) is becoming part of the clinical routine in AD, considering its ability to assess a broad range of these biomarkers and, therefore, of functional processes related to AD pathophysiology. PET imaging allows not only visual interpretation of the results for direct clinical use but, more importantly, provides quantitative brain data that may be used to further understand the pathophysiology [5].

The glucose analogue PET tracer 2-[^18^F]-fluoro-2-deoxy-D-glucose (FDG) can be used to assess alterations in brain glucose metabolism. In this way, clinical symptoms can optimally be linked to patterns of regionally impaired brain function, particularly when the symptoms cannot be explained by anatomical magnetic resonance imaging (MRI). This makes FDG PET a highly useful technique in the differential diagnosis of neurodegenerative diseases characterized by gradually emerging cognitive and/or motor defects [6]. Being one of the most accessible PET tracers in clinical routine, FDG PET has been extensively used in the early diagnosis and disease progression of AD [7]. However, it has become increasingly clear that AD is not expressed by a unique clinical syndrome [8,9], emphasizing the need for pathophysiological biomarkers, provided by e.g. ^11^C labelled Pittsburgh Compound B (PIB), and ^18^F-florbetaben PET. Classically, FDG PET studies have revealed that glucose metabolism is reduced in the parietal, temporal, posterior cingulate, and, less prominently, frontal cortices of AD patients when compared to healthy controls [5]. At the same time, visual analysis of amyloid PET data, with PET tracers such as PIB, provides information on the presence or absence of amyloid load in the brain, an aspect of major relevance in clinical practice in the early stages of AD [10]. When compared to healthy controls, AD patients showed increased PIB retention in cortical brain regions [11].

While complementary FDG and PIB PET scans might be helpful to improve diagnosis of AD, the use of different PET tracers increases costs, patient discomfort, scanning time, and exposure to radiation [12]. Therefore, the use of a single tracer that could provide information on more than one biomarker at the same time would be ideal. PIB PET is a well-known radiotracer for amyloid deposition [11], and due to its high lipophilicity, early-stage distribution in the brain might also be a good surrogate for brain perfusion [13,14]. These images can be assessed by making the time weighted average of the first frames of the dynamic PIB PET scan. The early frames provide an image that shows how the uptake of the tracer starts happening in the brain, and therefore can be used as a surrogate for brain perfusion. The time interval taken for PIB specifically has been optimized for the best correlation with FDG scans [15–18], but not for diagnostic purposes.

Moreover, compartmental models used for PIB PET quantification [19] also provide a good estimation of the rate constant for ligand transfer from blood to tissue (*K*_*1*_). Thus, *K*_*1*_ is expected to provide a good estimation of cerebral blood flow. An extensive study of non-invasive reference tissue based parametric methods established that the 2-step simplified reference tissue model (SRTM2) [20] allows a fast and easy computation of high-quality parametric images of relative tracer flow (*R*_1_) [21]. Since *R*_1_ represents the ratio between *K*_*1*_ from the tissue of interest and the reference region, it is probable that *R*_1_ parametric maps generated by the SRTM2 model provide a good image of relative cerebral blood flow (rCBF). Since regional glucose metabolism assessed with FDG PET and rCBF are closely related [22] in such a way that both are tightly coupled with regional neuronal activity [23,24], it enables the identification of regionally impaired brain function [25].

Therefore, the aim of the present study was to explore the use of *R*_1_, derived from dynamic PIB PET, as a surrogate for brain glucose metabolism, FDG PET, and then to examine if *R*_1_ estimates are more accurate than early-stage PIB uptake. To this end, regional *R*_1_ estimates obtained from voxel-based SRTM2 analysis of PIB PET scans were compared to the estimates of regional relative FDG and PIB early frames uptake in the same subjects.

## Material and methods

### Subjects

Thirty subjects were selected from a larger on-going study, recruited from 2013 till 2017 at the memory clinic of the University Medical Centre of Groningen (UMCG), Groningen, The Netherlands. The subjects included patients with either AD or MCI, and healthy controls. In all subjects, standard dementia screening was performed. Multimodal neuroimaging was also performed, including PIB and FDG PET scans, as well as T1-3D magnetic resonance imaging (MRI). Clinical diagnosis was established by consensus in a multidisciplinary team according to the National Institute on Aging Alzheimer’s Association criteria (NIA-AA) [26] for the AD patients, and for the MCI, the Petersen criteria [27]. Healthy subjects had no cognitive complaints, and a mini-mental state examination (MMSE) score above 28. Subjects were then classified into two categories based on visual inspection of the PIB PET images: "PIB+" (i.e. patients that presented high levels of cortical PIB binding), and "PIB-" (i.e. participants that presented low levels of cortical PIB). A summary of the demographic characteristics is shown in Table 1. All subjects provided written informed consent to participate in the study. Patients with a MMSE score higher than 18 were considered mentally competent to give informed consent. This cohort of subjects had a minimum MMSE score of 22, therefore all subjects were considered mentally competent to give informed consent. The study was conducted according to the Declaration of Helsinki and subsequent revisions. Ethical approval for the whole study, including the informed consent, was obtained from the Medical Ethical Committee of the UMCG (2014/320).

**Table 1:** Demographic summary of subjects.

Group		PiB+(*n* = 15)	PiB-(*n* = 15)	*p*-value
**Sex**	**Male**	10	10	
	**Female**	5	5	
**Diagnosis**	**AD**	8	1	
	**MCI**	7	3	
	**HC**	0	11	
**Age (y)**		66 ± 6	68 ± 4	0.20
**Weight (kg)**		78 ± 13	78 ± 13	0.81
**MMSE Score**		26 ± 3	29 ± 1	< 0.001

Demographic summary of the characteristics of the subjects included in this study. The p-values reported are resulted from a t-test comparing data from the PIB+ and PIB- groups.

### PET acquisition

All subjects underwent dynamic PIB PET and a static FDG PET examination with either a Siemens Biograph 40mCT or 64mCT PET/CT scanner (Siemens Medical Solutions, USA). A t-test comparing data provided by the different scanners showed that they yielded no statistically significant differences between them. Both scans were performed under standard resting conditions with eyes closed. The FDG PET scan was performed on the same day at least 90 minutes after the PIB injection for most subjects, while seven subjects had a delay of up to two months between scans. All subjects fasted for at least six hours before tracer injection, and plasma glucose levels were measured before the scan.

The PIB and FDG tracers were manufactured at the radiopharmacy facility at the Department of Nuclear Medicine and Molecular Imaging at the UMCG, synthesized accordingly to the Good Manufacturing Procedure, and administered via venous cannula. The dynamic PIB PET acquisition started at the moment of tracer injection (384 ± 48 MBq) and lasted for 60 minutes (frames: 7 × 10s, 3 × 30s, 2 × 60s, 2 × 120s, 2 × 180s, 5 × 300s, and 2 × 600s) for 17 of the 30 subjects, 70 minutes (one extra 600s frame) for 8 subjects, and 90 minutes (three 600s frames more than the 60 minutes scan) for 5 subjects. 20 minutes static FGD images were acquired 30 minutes after injection (207 ± 10 MBq). List-mode data from all PET scans were reconstructed using 3D OSEM (3 iterations and 24 subsets), point spread function correction and time-of-flight, resulting in images with 400 × 400 × 111 matrix, isotropic 2mm voxels, smoothed with 2mm Gaussian filter at Full Width and Half Maximum (FWHM).

### Image processing

Image registration and data analysis were done using PMOD software package (version 3.8; PMOD Technologies LLC). First, the T1 3D MRI was normalized to the Montreal Neurologic Institute space using tissue probability maps [28]. Then, the PET images were corrected for motion (in case of presence) by using the average of the first 12 frames as reference, and were aligned to the individual MRI. The Hammers atlas [29] was used to define anatomical brain volumes of interest (VOI), which were grouped on a total of 40 bilateral regions, with white matter separated from cortical tissue. Several regions from the original atlas were excluded from the analysis: cerebellar white matter, corpus callosum, third ventricle, lateral ventricle, and temporal horn. A list of regions used in this study is presented in S1 Table. Finally, the PET images were smoothed using a Gaussian filter of 6mm at FWHM, and everything that was outside of the brain was removed from the image.

Parametric images of rCBF (i.e., *R*_1_ images) were generated using the PIB PET images in the individual space and the SRTM2 [21]. The grey matter of the cerebellum was chosen as the reference since it is a region without relevant specific PIB binding [11,19,30,31]. To apply the model, a first estimate of the binding potential (*BP*_ND_) and efflux rate constant from the reference region (*k’*_*2*_) was obtained using the simplified reference tissue model (SRTM) [32]. Then, the *k’*_*2*_ parameter was defined as the median value from all voxels that have a BPnd value higher than 0.05. After that the *k’*_*2*_ parameter was fixed and the SRTM2 model was applied to generate the final *R*_1_ parametric images.

To compare with the early-stage distribution, data from the early frames of the PIB PET scans (ePIB) were generated using the time weighted average of the frames corresponding to different time intervals: 20s to 40s, 20s to 60s, 20s to 100s, 20s to 130s, and from 1min to 8min. In a previous study, it was shown that the interval from 1 to 8 minutes was the optimal interval when selecting early frames of PIB data, showing the best correlation with FDG uptake [15]. However, this time interval seems to be too long and might not be a pure measure of flow, but may already be presenting contributions from amyloid binding. Nonetheless, this interval was also considered, along with other earlier times that corresponded better with the influx of the tracer into the brain. After the weighted averages were estimated, standardized uptake values were calculated and then normalized to the values of the reference region (cerebellum grey matter) for the same time interval.

To compare PIB derived *R*_1_ and ePIB to FDG PET, standardized uptake value ratios (SUVR) were generated for the FDG PET images by normalizing the uptake to the mean value of the cerebellum (grey matter). All images were also transformed to atlas space for further voxel-based comparison. To better visualize the differences between the images, the FDG SUVR images were subtracted from the *R*_1_ and ePIB for each subject, and the mean image of all subjects per group was generated. This was done for an easier visual comparison voxel-by-voxel of the differences between the images.

Finally, the images were also corrected for partial volume effects using the geometric transfer matrix method [33] to explore the possible impact of brain atrophy in the results.

### Statistics

A general linear model was used to explore the relationship between *R*_1_ or ePIB (dependent variable) and FDG SUVR (independent variable) estimates of all regions for the subjects of each group. Additionally, in order to compare how much the suggested methods correlate, the same analysis was made between ePIB (dependent) and *R*_1_ (independent variable). A *p*-value of 0.05 was used as significance threshold for all analyses.

A Bland-Altman plot was made to evaluate the agreement between the two measurements (*R*_1_ or ePIB and SUVR). The difference between the two was plotted against the average SUVR values per region, considering the FDG PET measure as the reference [34]. All statistical analyses were done using Rstudio (R version 3.4.0, [35] Rstudio version 1.0.143).

To compare the capacity of the methods to distinguish between PIB+ and PIB- groups, a voxel-based comparison was performed using SPM12 (Wellcome Trust Centre for Neuroimaging, UK). A two-sample t-test between the groups was done independently for FDG SUVR, *R*_1_, and ePIB images. Interpretation of the resulting T-maps was done using a voxel threshold of *p* = 0.005 (uncorrected), and only clusters with *p* < 0.05 corrected for family-wise error were considered significant.

## Results

### Group differences

**Fig 1:**
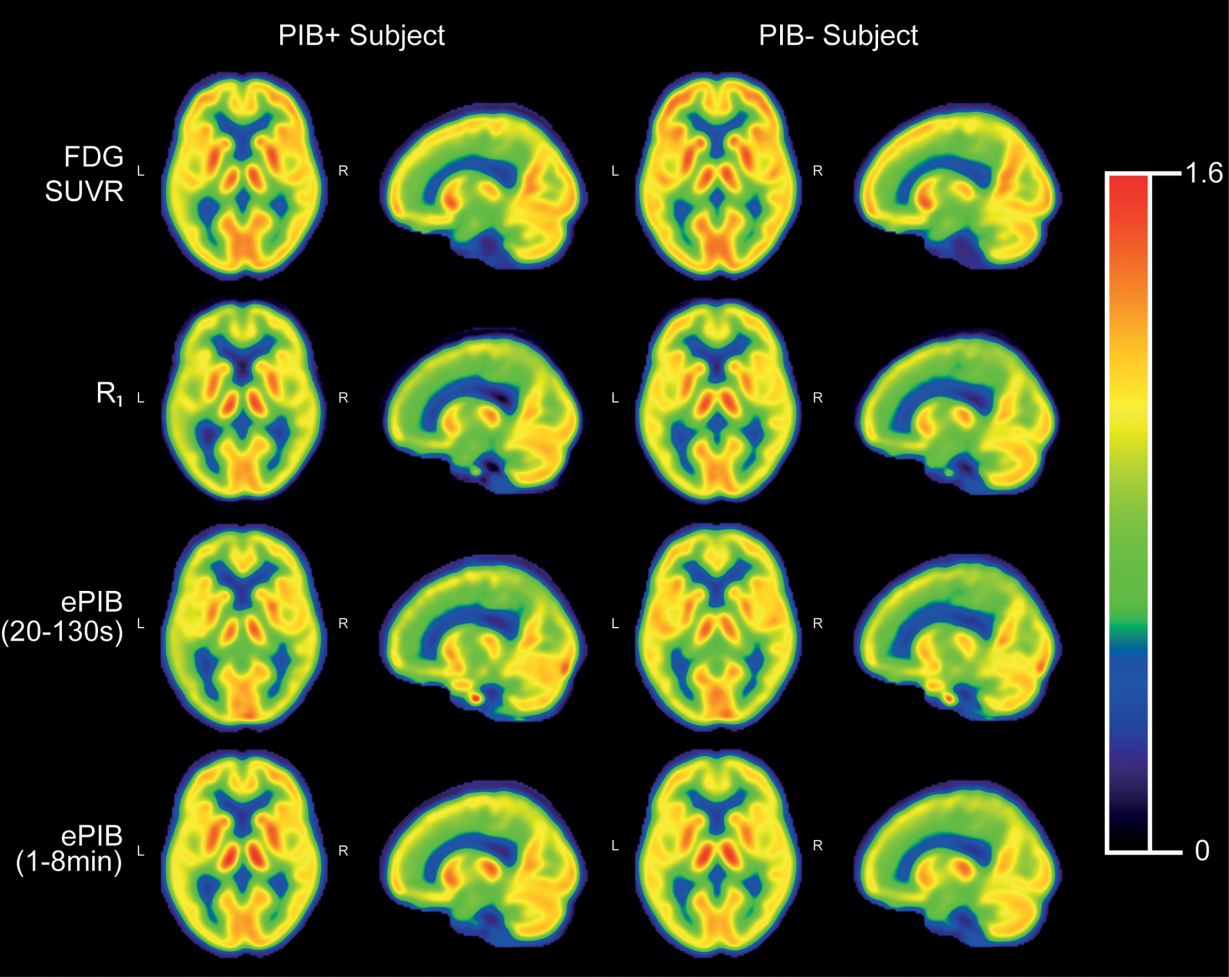
Mean images per group. Mean normalized FDG uptake images (first row), parametric images of PiB rCBF (*R*_1_; second row), and time weighted average of early PIB frames (20 to 130 seconds, and 1 to 8 minutes; third and fourth row, respectively) of the PIB+ group (left), and the PIB- group (right). Shown are corresponding transaxial, and sagittal slices of the brain. All colour scales were adjusted to the same range.

In general terms, the FDG SUVR images were in agreement with the literature (Fig 1) [5]. In the images from the PIB+ group, disease patterns of regional hypometabolism corresponded to those from literature [36]. The PIB- group did not show abnormally decreased cortical uptake. The resemblance between the *R*_1_ and FDG SUVR images was noticeable, with similar AD specific patterns in the PIB+ group. Better visual similarity between ePIB, SUVR, and *R*_1_ was only seen for later intervals of frames. Representative images for all time intervals can be seen in S1 Fig.

**Fig 2:**
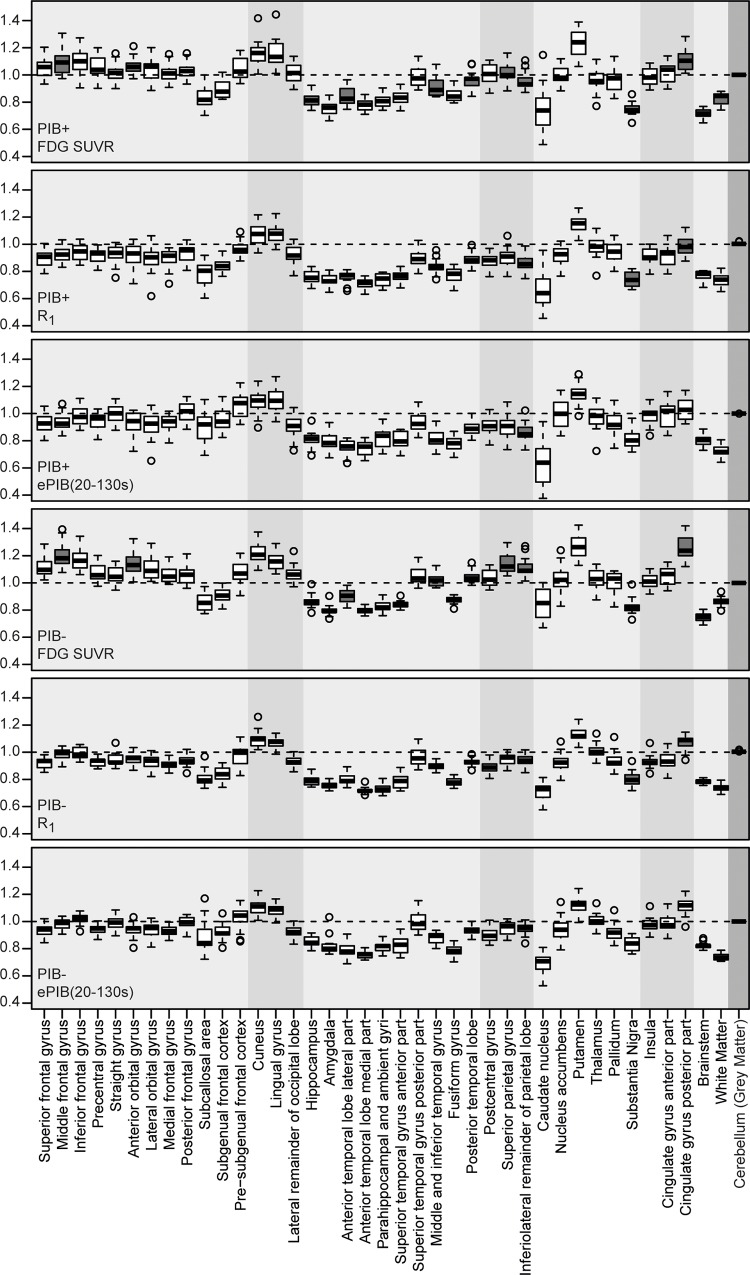
Distribution of *R*_1_ and FDG SUVR values per region. Distribution of individual subject's FDG SUVR (first and fourth rows), *R*_1_ (second and fifth rows), and ePIB(20-130s) (third and sixth rows) values per group in all VOIs. First three rows contain the values from the PIB+ group while the last three, from the PIB- group. Filled boxes represent regions that showed a statistical significant difference between PIB+ and PIB- groups. The filling of the background was made to differentiate between brain areas: frontal lobe, occipital lobe, temporal lobe, parietal lobe, central structures, insula and cingulate gyri, posterior fossa, white matter, and grey matter of the cerebellum (reference region), in this order.

The FDG SUVR values from each region across subjects were (mean ± SD) 0.96 ± 0.15 (range 0.49–1.44) and 1.01 ± 0.15 (0.67–1.42) for the PIB+ and PIB- patients respectively. Typical regions known for reduced FDG uptake such as the frontal and parietal lobes presented relative uptake values of 1.01 ± 0.11 (PIB+) and 1.07 ± 0.12 (PIB-) for the frontal cortex, and of 0.99 ± 0.08 and 1.10 ± 0.09 for the parietal cortex (Fig 2). The regions that presented the highest FDG SUVR mean values were the putamen (1.23 ± 0.10), for the PIB+ group, and the cingulate gyrus posterior part along with the putamen (1.26 ± 0.09) for the PIB- group. Meanwhile, the brainstem was the region that presented the lowest values for both groups (0.72 ± 0.03 and 0.75 ± 0.03 for the PIB+ and PIB- groups respectively).

For the *R*_1_ data, the regional values were 0.88 ± 0.13 (range 0.46–1.27) for the PIB+ group, and 0.90 ± 0.11 (0.58–1.26) for the PIB-. Frontal cortex presented uptake values of 0.90 ± 0.08 (PIB+) and 0.93 ± 0.07 (PIB-), while for the parietal cortex, these values were 0.88 ± 0.07 and 0.93 ± 0.05, respectively (Fig 2). The region that presented the higher values was, for both groups, the Putamen (1.14 ± 0.07 for the PIB+ group and 1.13 ± 0.05 for the PIB- group). The smallest R1 values were found on the caudate nucleus, for both groups (0.66 ± 0.14 and 0.71 ± 0.07 respectively for the PIB+ and PIB- groups).

The values for ePIB(20-130s) across all subjects were 0.91 ± 0.13 (range 0.38–1.29) and 0.93 ± 0.11 (0.53–1.24) for the PIB+ and PIB- groups respectively. Frontal cortex presented values of 0.96 ± 0.09 (0.65–1.23) for PIB+ patients, and 0.96 ± 0.07 (0.72–1.17) for PIB- subjects. Meanwhile, the parietal cortex had values of 0.89 ± 0.08 (0.73–1.09), and 0.93 ± 0.05 (0.83–1.02) for PIB+ and PIB- subjects respectively. The putamen was the region that presented the highest values of ePIB(20-130s) (1.14 ± 0.08 for the PIB+ group and 1.12 ± 0.06 for the PIB-). Meanwhile, the caudate nucleus presented the lowest values (0.64 ± 0.17 and 0.69 ± 0.07 for the PIB+ group and for the PIB- group respectively).

With the exception of the hippocampus, brainstem, and white matter, all other regions in all methods presented higher average values after the partial volume effect correction was applied. For the FDG SUVR and the *R*_1_ methods, the thalamus also yielded lower values than the other regions. In general, the number of regions that presented a statistically significant difference between groups was reduced. Moreover, the standard deviations of the data increased after partial volume correction.

A summary of all results for each region of both groups can be found in S2–S8 Tables, and the results from the partial volume corrected data for the FDG SUVR, *R*_1_, ePIB(20-130s)m and ePIB(1-8min) methods are shown in S9–S12 Tables.

### Difference images

**Fig 3:**
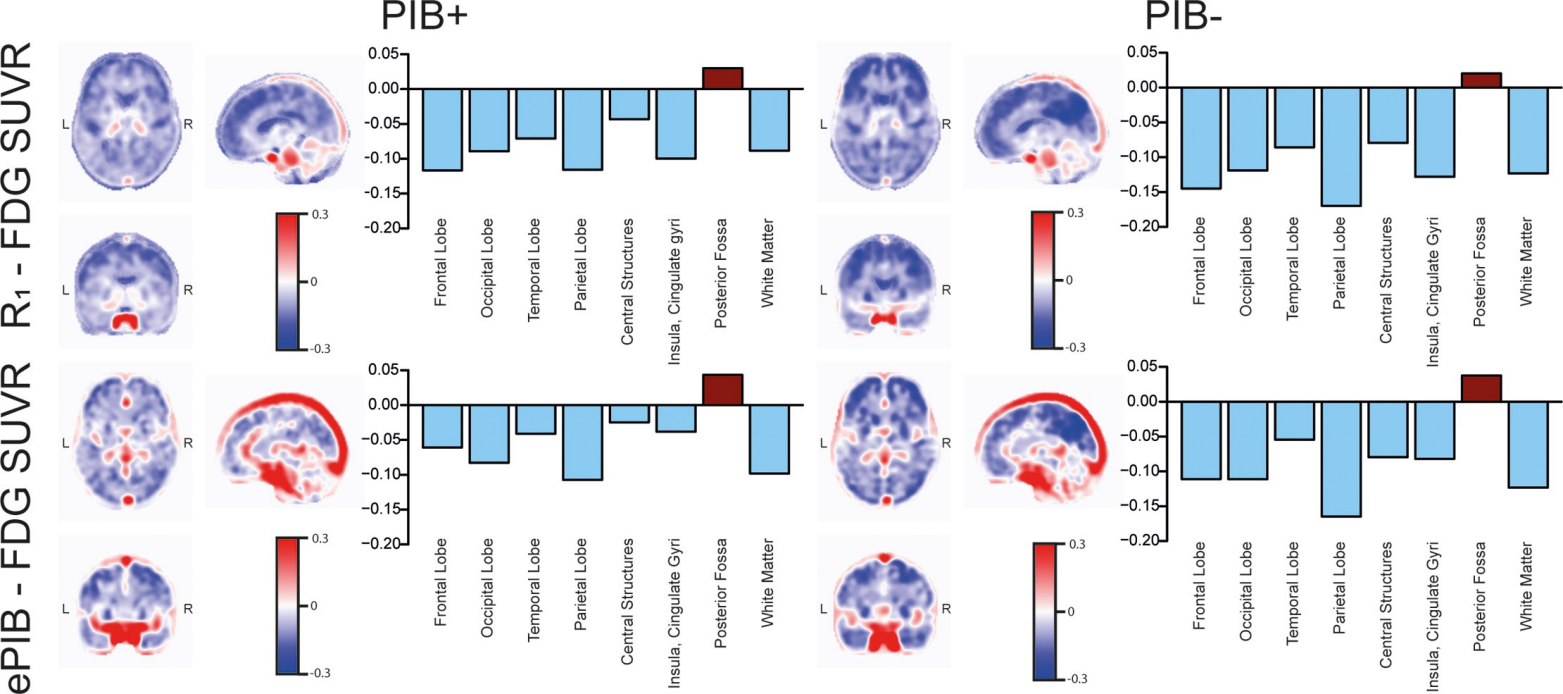
Mean difference images per group. Mean difference images per groups comparing normalized FDG uptake and *R*_1_ parametric maps (*R*_1_ –FDG SUVR; first row), and FDG SUVR and ePIB(20 to 130 seconds) images (ePIB–FDG SUVR; second row). On the left, the mean difference image for the PIB+ group can be seen, and on the right, the PIB-. The closer the *R*_1_ or ePIB and FDG SUVR estimates, the more white the voxel appears. Negative values correspond to voxels where the FDG SUVR voxel presented a higher value than the *R*_1_ or ePIB. Bar plots representing the mean values of brain areas for each group are on the right of the respective images.

When exploring the mean difference images per group (Fig 3), a clear difference was observed between PIB+ and PIB- groups. Overall, higher FDG SUVR values were found than those of *R*_1_ and ePIB (blue colour, Fig 3) especially at the parietal and frontal lobes of the PIB- subjects. Structures such as the thalamus, brainstem, and the cerebellum showed the opposite pattern, with higher *R*_1_ or ePIB values than FDG SUVR (red colour, Fig 3). The difference images for all time intervals included in this study can be seen in S2 Fig.

### Correlation between methods and SUVR

**Fig 4:**
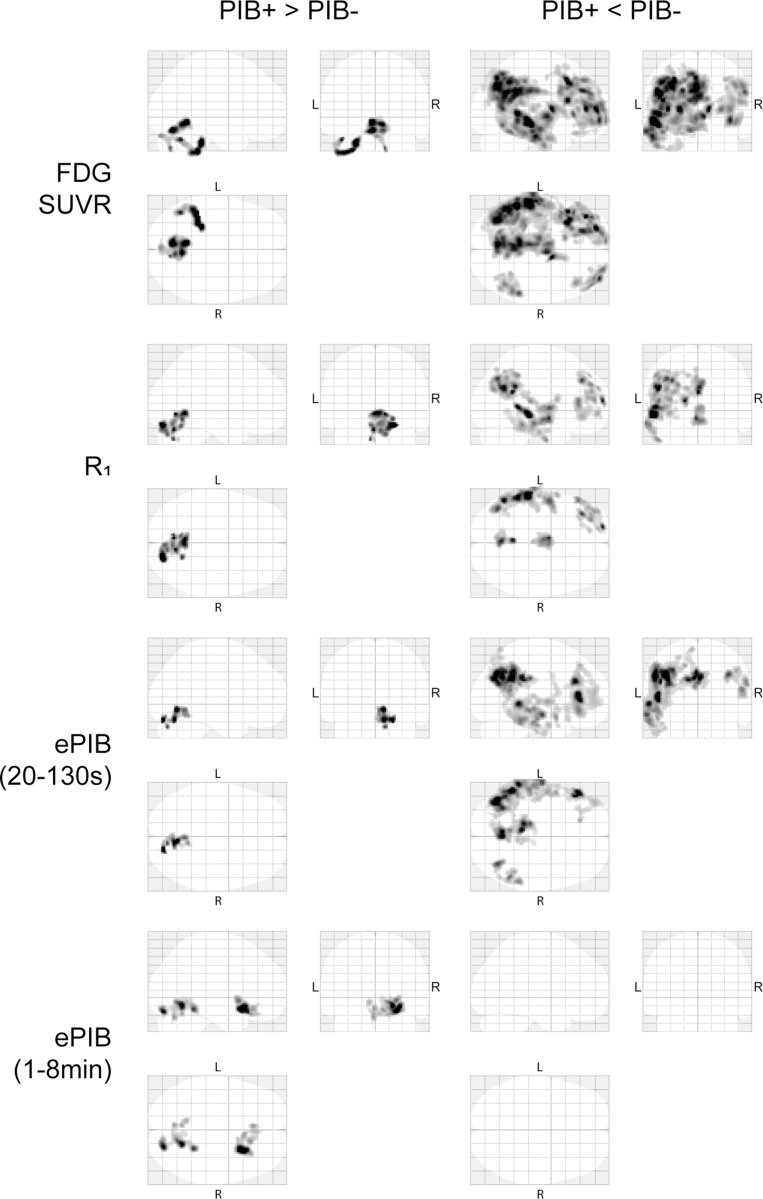
Linear regression analysis for R_1_ and ePIB(20-130s) estimates. Scatter plot showing regional CBF estimates from R_1_ parametric images (top) and ePIB(20-130s; bottom) (y-axis), and normalized FDG FDG uptake (x-axis). Data are arranged according to patient group: circles represent PIB+ group, and triangles PIB-. Lines resulting from the linear regression applied to the data are also shown: a full line for the PIB+ group, and a dashed one for PIB-. Results of the linear regression are given in boxes at the bottom right corner.

The scatter plots of rCBF estimates from *R*_1_ and FDG SUVR uptake (Fig 4) suggest a strong correlation between *R*_1_ and FDG SUVR values, 0.86 for the PIB+ group and 0.84 for the PIB-. In addition, FDG SUVR estimates were highly predictive of *R*_1_, accounting for 72% (*R*^2^ = 0.72, *p* < 0.001, slope = 0.73, intercept = 0.18) and 67% (*R*^2^ = 0.67, *p* < 0.001, slope = 0.62, intercept = 0.27) of its variability, for PIB+ and PIB- respectively. While ePIB(20-130s) also presented a good correlation when compared to FDG SUVR, of 0.79 (PIB+) and 0.73 (PIB-), this method was not as predictive as *R*_1_, accounting for 62% (*R*^2^ = 0.62, *p* < 0.001, slope = 0.73, intercept = 0.21) for the PIB+ patients, and 54% (*R*^2^ = 0.54, *p* < 0.001, slope = 0.56, intercept = 0.36) for the PIB- subjects. S3 Fig depicts the same data but with subjects divided by diagnosis instead of PIB uptake.

### Bias assessment

**Fig 5:**
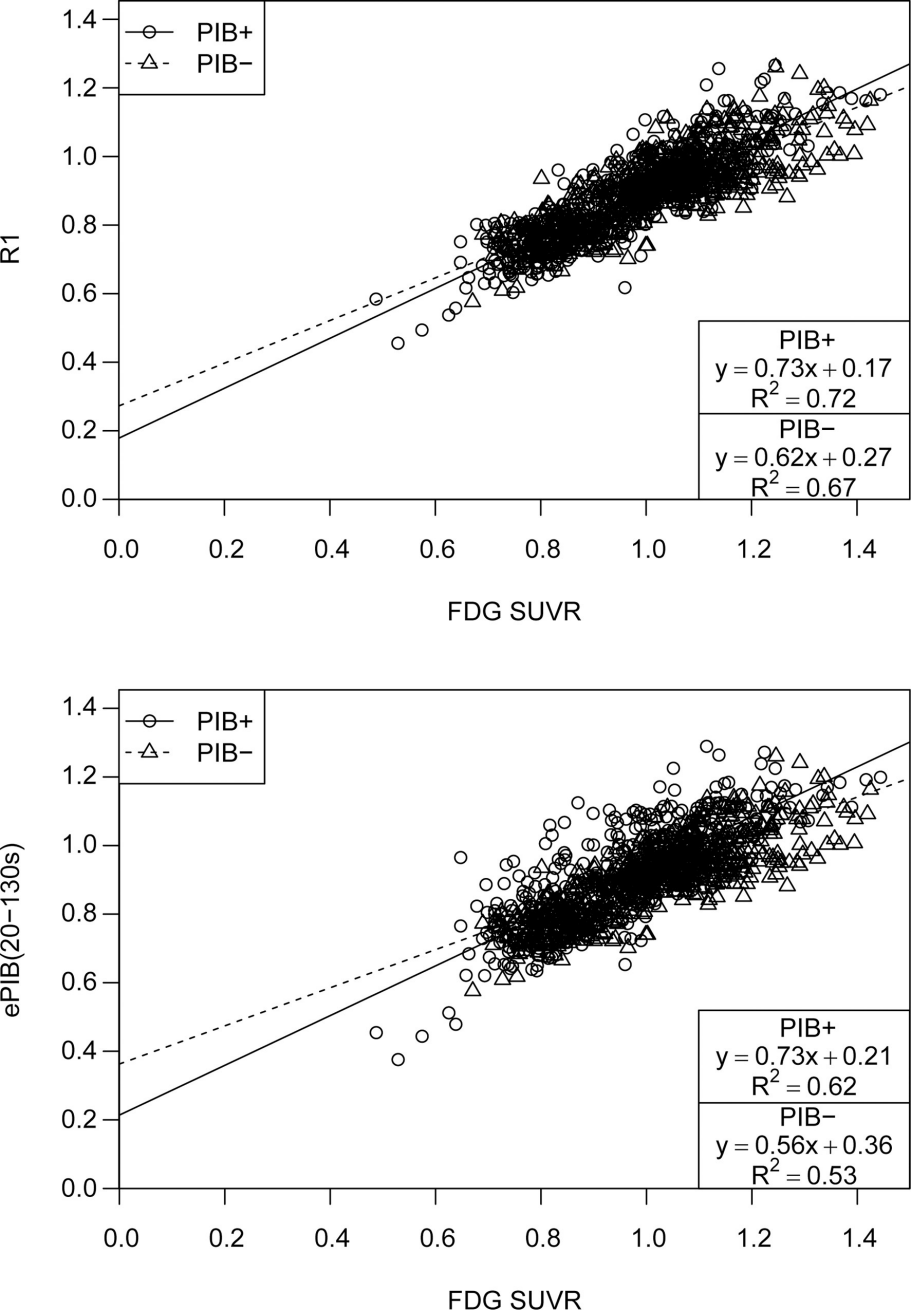
Bland-Altman plot. Bland-Altman plot showing the difference between the values of rCBF assessed by different methods (by *R*_1_, on the top row, and by ePIB(20 to 130 seconds), on the bottom, estimations and from the normalized FDG uptake). Circles represent data from the PIB+ group, while triangles represent PIB-. The full line is at the mean difference value for all data (not classified in groups), and the dashed lines delimit the 95% agreement interval (at mean ± 1.96 × standard deviation).

Moreover, the mean bias between *R*_1_ and FDG SUVR (Fig 5) was of -0.08 ± 0.08 (range -0.34–0.13) and -0.11 ± 0.09 (-0.39–0.13) for the PIB+ and PIB- groups, respectively. A moderate negative trend, proportional to the magnitude of the SUVR estimate was also observed. A linear regression from this data showed a bias of 27% for the PIB+ group (*R*^2^ = 0.27, *p* < 0.001, slope = -0.27, intercept = 0.18), and of 43% for the PIB- group (*R*^2^ = 0.43, *p* < 0.001, slope = -0.38, intercept = 0.27). Furthermore, ePIB(20-130s) presented a similar negative trend, with a mean of -0.05 ± 0.09 (range -0.31–0.32) for the PIB+ group, and -0.09 ± 0.10 (-0.39–0.30) for the PIB-. For the PIB+ patients, a bias of 19% (*R*^2^ = 0.19, *p* < 0.001, slope = -0.27, intercept = 0.19) was found, while the PIB- subjects presented a bias of 42% (*R*^2^ = 0.42, *p* < 0.001, slope = -0.44, intercept = 0.36).

### Correlation between ePIB and *R*_1_

When comparing ePIB with *R*_1_, later time frames (20s to 100s, 20s to 130s, and 1min to 8 min) presented the best correlations and predictability. Results are shown in Table 2. While those intervals showed no significant bias for the PIB+ group, for the PIB- subjects, there was a slight negative bias of 4%, 3%, and 22% respectively.

**Table 2:** Linear regression results of the analysis between ePIB and *R*_1_.

		PIB+	PIB-
**20-40s**	***R*^2^**	0.00	0.00
	**Intercept**	0.33	1.01*
	**Slope**	1.13	-0.07
**20-60s**	***R*^2^**	0.03*	0.01*
	**Intercept**	0.56*	0.83*
	**Slope**	0.61*	0.33*
**20-100s**	***R*^2^**	0.65*	0.55*
	**Intercept**	0.11*	0.21*
	**Slope**	0.95*	0.84*
**20-130s**	***R*^2^**	0.87*	0.87*
	**Intercept**	0.03*	0.08*
	**Slope**	1.00*	0.93*
**1-8min**	***R*^2^**	0.94*	0.96*
	**Intercept**	0.04*	0.12*
	**Slope**	0.90*	0.90*

Results from the linear regression comparing the data from the *R*_1_ parametric maps and the early frames images.

*p <0.05

### Voxel-based comparison between groups

**Fig 6:**
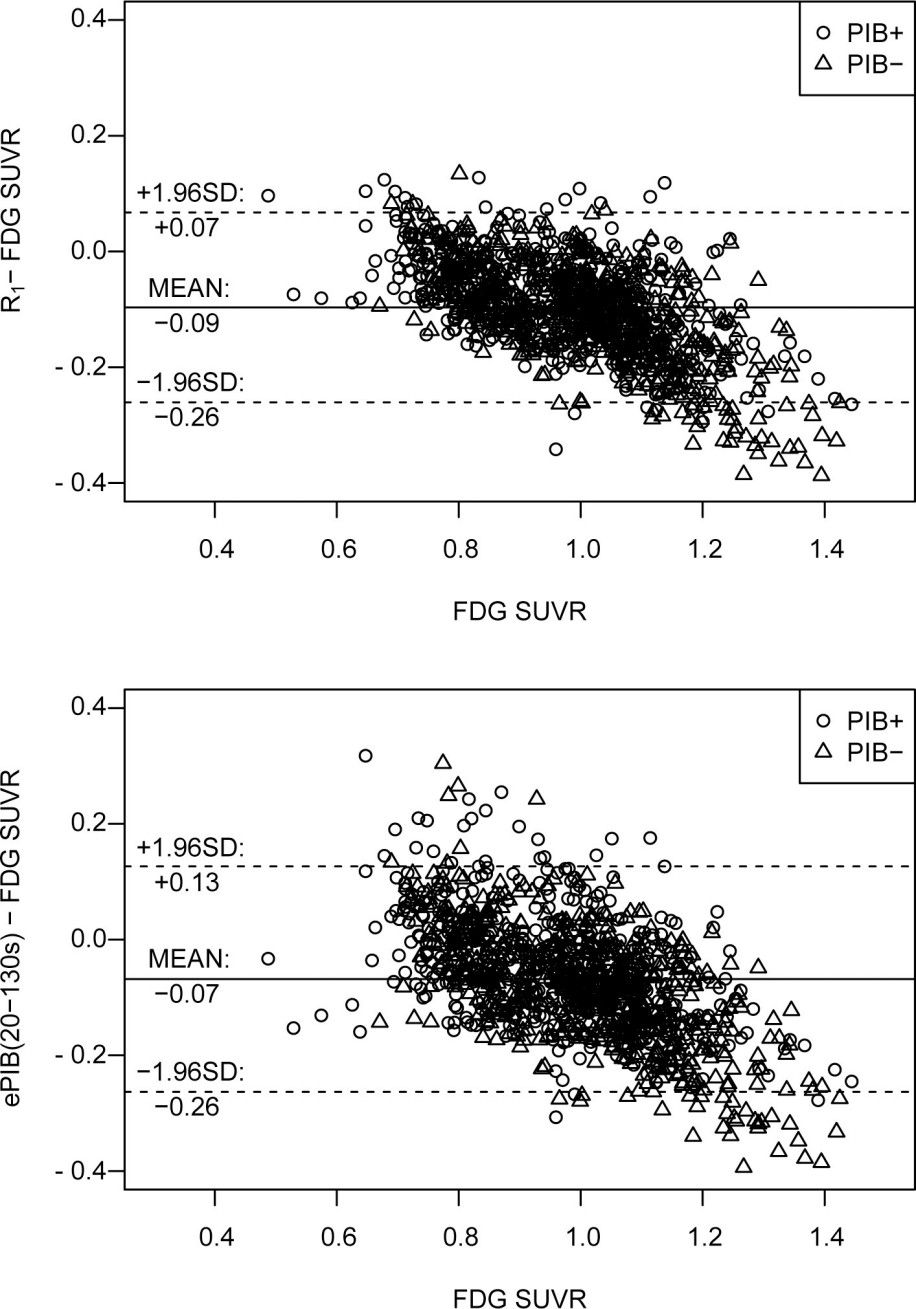
SPM analysis. Maximum Intensity Projections derived from the voxel based analysis. First row contains the images from FDG SUVR, second row shows *R*_1_, third, ePIB(20 to 130 seconds), and fourth, ePIB(1 to 8 minutes). On the left, statistically significant regions where PIB+ group shows higher rCBF than the PIB- group, and, on the right, statistically significant regions where the PIB- group showed higher flow than the PIB+ group.

Voxel-based analysis showed statistically significant differences between subject groups in FDG SUVR and *R*_1_ images. The main discrepancies between tracers were the size of the clusters: while FDG SUVR had larger clusters, their main core could also be seen in *R*_1_ (Fig 6). When exploring the differences between groups where PIB- subjects showed a higher flux than PIB+ subjects, the clusters were present in frontal, temporal, and parietal regions of both tracers. Meanwhile, when exploring the opposite differences, the brainstem and part of the cerebellum were the only visible region.

The same analysis was done for all ePIB intervals. The shorter intervals (20s to 40s, and 20s to 60s) showed no statistically significant clusters. Meanwhile, the 20s to 130s interval presented similar results as *R*_1_ and FDG SUVR analysis also being able to display cluster that showed regions of PIB+ patients that had a larger perfusion than PIB- subjects. Later intervals (1 to 8 minutes) were not able to produce the same pattern of distinction between groups (Fig 6).

## Discussion

In this study, the possibility of using PIB *R*_1_ and ePIB as surrogates for FDG SUVR was explored. The good agreement between measures and the resemblance of the images indicate that rCBF estimates from pharmacokinetic analysis of PIB might be a good alternative to an additional FDG scan. Therefore, a single PIB PET study might suffice to observe amyloid deposition and assess rCBF, as a surrogate for the FDG. This approach bypasses some limitations associated with PET studies and facilitates dual biomarker imaging, as subjects only need to undergo one scan, thus reducing radiation exposure and costs. For the estimation of the *R*_1_ in the present study, the SRTM2 model was used since it has been previously validated as the most suitable reference tissue model for PIB [21]. Although earlier studies indicate that PIB tissue kinetics are best represented by a two-tissue compartment [11], the resulting bias on the *BP*_ND_ using SRTM is small [37]. Optimization of *k’*_*2*_ estimation has also been made [20,38], but it has been observed that *R*_1_ is insensitive to minor inaccuracies of that estimation [32]. Taken together, this strongly suggests that SRTM2 might be a suitable choice for the *R*_1_ estimation.

The results presented in the previous section showed a high correlation between *R*_1_ estimates and FDG SUVR values. This response was already anticipated, as *R*_1_ has been found to be a valid marker of rCBF [39], and there is an established coupling between blood flow delivery and metabolic demand [23]. The high predictability of FDG SUVR by *R*_1_ (about 72%) also indicates that *R*_1_ might be a good surrogate for FDG PET imaging.

Several studies have presented early frames from PIB [2,15,16,40] and other Aβ tracers [18,41–43] as a proxy to FDG. But while their choice of time interval was solely done considering the best correlation with FDG SUVR values, this study also considered the bias between measurements and how well the method could differentiate between the PIB+ and PIB- groups on a voxel level. The recommended time interval of 1 to 8 minutes [15] was not able to distinguish between the PIB+ and PIB- groups, and showed a similar bias to the measure when compared to FDG SUVR. This was of interest considering that, in a previous study [15], this time frame showed the best correlation with the FDG SUVR values. This time interval might be too long and thus picking up some amyloid binding. This would affect the results since it might not be a pure flow image. Considering these results, the early frames from 1 to 8 minutes may not be the best time interval to estimate regional flow distributions. Therefore, the preferred time interval taken here was from 20 to 130 seconds. Although the good resemblance of the images and the good correlation with both *R*_1_ and FDG SUVR measures, *R*_1_ still presented better correlation with FDG SUVR than ePIB. This might be due to the non-uniform distribution of the tracer, small sampling window, and noise [15]. Therefore, the use of a full kinetic modelling approach might be a better approach, since it not only provides an estimation of amyloid deposition through *BP*_ND_ [2], but also an estimation of rCBF through *R*_1_.

Moreover, the voxel-based group comparison demonstrated that *R*_1_ allows identification of regions with reduced rCBF in the PIB+ group, as compared with PIB-. This pattern coincided with the distribution of reduced in glucose metabolism, although cluster sizes appear to be smaller with *R*_1_ than with FDG SUVR (Fig 6). Again, these results suggest that changes in *R*_1_ distribution between subject groups seem to approximate those of FDG SUVR reasonably well, but possibly at the cost of lower sensitivity. Therefore, the diagnostic performance of using *R*_1_ parametric maps as a surrogate of FDG SUVR for diagnosis of AD will be further explored, although it can already be said that it might not be the best option for longitudinal studies due to its lower sensitivity to small changes. This sensitivity to differences between groups was also reduced when performing partial volume correction which is caused by an increment in data variance. This increased variance might be due to a higher noise, as consequence of the correction; an increased variability between subjects due to errors in the segmentation of tissues; or it might be a real larger spread in values. Moreover, previous studies have found that partial volume correction might lead to bigger bias on results[44,45], and there is not clear consensus about its use.

Despite the fact that these findings showed a good correlation between *R*_1_ and SUVR values, some differences were also observed in the data. The slopes of the linear regressions were smaller than one, suggesting that *R*_1_ might be less sensitive than FDG SUVR. Moreover, the bias between *R*_1_ and FDG SUVR seems to increase with higher FDG SUVR values (Fig 5). In addition, FDG SUVR values were higher than those of *R*_1_ for most of the brain regions, with the exception of the cerebellum, brainstem, and thalamus (Fig 3). These regions are known to be hyperperfused due to their “potential need for precipitous and rapid activation” [46]. Therefore, *R*_1_ should not be used for studies of changes in metabolism and pathophysiology, since it is not a true surrogate of FDG, but it can be considered for (differential) diagnosis.

Interestingly, the degree of agreement between *R*_1_ and FDG SUVR values seems to be different for the PIB+ and the PIB- groups. Deposition of Aβ induces profound changes in the neurons' metabolic phenotype [24] and it is known to relate with neurovascular decoupling, resulting in a cerebrovascular dysfunction with a reduction of the rCBF [47]. For this study, it was hypothesized that the agreement between *R*_1_ and FDG SUVR images would be worse in the PIB+ group, where FDG will capture not only alterations in perfusion, but also in metabolism. However, what was found seems to be in contradiction with this initial hypothesis. The fact that PIB+ group presented a better correlation than the PIB-, a lower bias, and a higher predictability of FDG SUVR values might suggest that the *R*_1_ (i.e. PIB PET) could more accurately replace FDG PET in PIB+ subjects than in the PIB-.

## Conclusion

Phamacokinetic analysis of dynamic PIB PET studies provides high-quality rCBF images comparable with those obtained by FDG SUVR. The high correlation between *R*_1_ and normalized FDG uptake suggests that PIB PET parametric maps might be used as an alternative to FDG PET, and also *R*_1_ outperformed ePIB in this analysis. However, despite the good correlation between *R*_1_ and SUVR, there is still a need for further prospective studies to validate the use of *R*_1_ as an alternative of FDG SUVR for diagnostic purposes, and to monitor the progression of AD, since the observed differences between *R*_1_ and FDG SUVR might be of importance in longitudinal studies, for example, where small effect sizes are relevant. Nevertheless, the results presented in this study suggest that *R*_1_ might be used as a surrogate for FDG and preferred over ePIB for (differential) diagnosis in neurodegenerative diseases with PET imaging.

## Supporting information

S1 Fig Representative studies. Representative images of normalized FDG uptake images (first row), parametric images of PiB rCBF (*R*_1_; second row), and all time weighted average of early PIB frames (20 to 40 seconds on the third row, 20 to 60 seconds on the fourth, 20 to 100 seconds on the fifth, 20 to 130 seconds on the sixth, and 1 to 8 minutes on the seventh row) of a PIB+ patient (left), and a PIB- subject (right). Shown are corresponding transaxial, and sagittal slices of the brain. All colour scales were adjusted to the same range.
(TIF)

S2 Fig Mean difference images per group. Mean difference images per groups comparing normalized FDG uptake and *R*_1_ parametric maps (*R*_1_ –SUVR; first row), and for all ePIB time intervals: 20 to 40 seconds (second row), 20 to 60 seconds (third row), 20 to 100 seconds (fourth row), 20 to 130 seconds (fifth row), and 1 to 8 minutes (sixth row). On the left, the mean difference image for the PIB+ group can be seen, and on the right, the PIB-. The closer the rCBF and SUVR estimates, the more white the voxel appears. Negative values correspond to voxels where the SUVR voxel presented a higher value than the *R*_1_ or ePIB.
(TIF)

S3 Fig Linear regression of subjects separated by diagnosis. Scatter plot showing regional CBF estimates from R_1_ parametric images (top) and ePIB(20-130s; bottom) (y-axis), and normalized FDG uptake (x-axis). Data are arranged according to subject diagnosis: red points represent AD patients, green points represent the HC subjects, and blue points represent MCI participants. Lines resulting from the linear regression applied to the data are also shown: a full line for the AD group, a dashed one for HC subjects, and a dot and dash line for the MCI participants. Results of the linear regression are given in boxes at the bottom right corner.
(TIF)

S4 Fig SPM analysis of all methods. Maximum Intensity Projections derived from the voxel based analysis. The rows contain, in order from top to bottom, FDG SUVR, *R*_1_, ePIB(20-40s), ePIB(20-60s), ePIB(20-100s), ePIB(20-130s), and ePIB(1-8min). On the left, statistically significant regions where PIB+ group shows higher rCBF than the PIB- group, and, on the right, statistically significant regions where the PIB- group showed higher flow than the PIB+ group.
(TIF)

S1 Table Regions of interest. List of all regions of interest included and their separation in this study.
(DOCX)

S2 Table FDG SUVR values. FDG SUVR values (expressed as mean ± standard deviation) for each region per subject group, and uncorrected and corrected for false discovery rate p-values from the t-test.
(DOCX)

S3 Table *R*_1_ values. *R*_1_ values (expressed as mean ± standard deviation) for each region per subject group, and uncorrected and corrected for false discovery rate p-values from the t-test.
(DOCX)

S4 Table ePIB(20-40s) values. ePIB(20-40s) values (expressed as mean ± standard deviation) for each region per subject group, and uncorrected and corrected for false discovery rate p-values from the t-test.
(DOCX)

S5 Table ePIB(20-60s) values. ePIB(20-60s) values (expressed as mean ± standard deviation) for each region per subject group, and uncorrected and corrected for false discovery rate p-values from the t-test.
(DOCX)

S6 Table ePIB(20-100s) values. ePIB(20-100s) values (expressed as mean ± standard deviation) for each region per subject group, and uncorrected and corrected for false discovery rate p-values from the t-test.
(DOCX)

S7 Table ePIB(20-130s) values. ePIB(20-130s) values (expressed as mean ± standard deviation) for each region per subject group, and uncorrected and corrected for false discovery rate p-values from the t-test.
(DOCX)

S8 Table ePIB(1-8min) values. ePIB(1-8min) values (expressed as mean ± standard deviation) for each region per subject group, and uncorrected and corrected for false discovery rate p-values from the t-test.
(DOCX)

S9 Table FDG SUVR partial volume corrected values. FDG SUVR values corrected for partial volume effects (expressed as mean ± standard deviation) for each region per subject group, and uncorrected and corrected for false discovery rate t-values from the t-test.
(DOCX)

S10 Table *R*_1_ partial volume corrected values. *R*_1_ values corrected for partial volume effects (expressed as mean ± standard deviation) for each region per subject group, and uncorrected and corrected for false discovery rate t-values from the t-test.
(DOCX)

S11 Table ePIB(10-130s) partial volume corrected values. ePIB(20-130s) values corrected for partial volume effects (expressed as mean ± standard deviation) for each region per subject group, and uncorrected and corrected for false discovery rate t-values from the t-test.
(DOCX)

S12 Table ePIB(1-8min) partial volume corrected values. ePIB(1-8min) values corrected for partial volume effects (expressed as mean ± standard deviation) for each region per subject group, and uncorrected and corrected for false discovery rate t-values from the t-test.
(DOCX)

## References

[1] Jack CR, Knopman DS, Jagust WJ, Shaw LM, Aisen PS, Weiner MW, et al Hypothetical model of dynamic biomarkers of the Alzheimer’s pathological cascade. Lancet Neurol. 2010;9: 119–128. 10.1016/S1474-4422(09)70299-6 20083042 PMC2819840

[2] Meyer PT, Hellwig S, Amtage F, Rottenburger C, Sahm U, Reuland P, et al Dual-Biomarker Imaging of Regional Cerebral Amyloid Load and Neuronal Activity in Dementia with PET and 11C-Labeled Pittsburgh Compound B. J Nucl Med. 2011;52: 393–400. 10.2967/jnumed.110.083683 21321269

[3] Gomperts SN, Rentz DM, Moran E, Becker JA, Locascio JJ, Klunk WE, et al Imaging amyloid deposition in lewy body diseases. Neurology. 2008;71: 903–910. 10.1212/01.wnl.0000326146.60732.d6 18794492 PMC2637553

[4] Rowe CC, Ng S, Ackermann U, Gong SJ, Pike K, Savage G, et al Imaging beta-amyloid burden in aging and dementia. Neurology. 2007;68: 1718–25. 10.1212/01.wnl.0000261919.22630.ea 17502554

[5] Nordberg A, Rinne JO, Kadir A, Långström B. The use of PET in Alzheimer disease. Nat Rev Neurol. Nature Publishing Group; 2010;6: 78–87. 10.1038/nrneurol.2009.217 20139997

[6] Teune LK, Bartels AL, De Jong BM, Willemsen ATM, Eshuis SA, De Vries JJ, et al Typical cerebral metabolic patterns in neurodegenerative brain diseases. Mov Disord. 2010;25: 2395–2404. 10.1002/mds.23291 20669302

[7] Minoshima S, Giordani B, Berent S, Frey KA, Foster NL, Kuhl DE. Metabolic reduction in the posterior cingulate cortex in very early Alzheimer’s disease. Ann Neurol. 1997;42: 85–94. 10.1002/ana.410420114 9225689

[8] Jagust W, Reed B, Mungas D, Ellis W, DeCarli C. What does fluorodeoxyglucose PET imaging add to a clinical diagnosis of dementia? Neurology. 2007;69: 871–877. 10.1212/01.wnl.0000269790.05105.16 17724289

[9] Mosconi L. Brain glucose metabolism in the early and specific diagnosis of Alzheimer’s disease: FDG-PET studies in MCI and AD. Eur J Nucl Med Mol Imaging. 2005;32: 486–510. 10.1007/s00259-005-1762-7 15747152

[10] Grimmer T, Henriksen G, Wester HJ, Förstl H, Klunk WE, Mathis CA, et al Clinical severity of Alzheimer’s disease is associated with PIB uptake in PET. Neurobiol Aging. 2009;30: 1902–1909. 10.1016/j.neurobiolaging.2008.01.016 18346821

[11] Klunk WE, Engler H, Nordberg A, Wang Y, Blomqvist G, Holt DP, et al Imaging brain amyloid in Alzheimer’s disease with Pittsburgh Compound-B. Ann Neurol. 2004;55: 306–19. 10.1002/ana.20009 14991808

[12] Teipel S, Drzezga A, Grothe MJ, Barthel H, Chételat G, Schuff N, et al Multimodal imaging in Alzheimer’s disease: Validity and usefulness for early detection. Lancet Neurol. 2015;14: 1037–1053. 10.1016/S1474-4422(15)00093-9 26318837

[13] Blomquist G, Engler H, Nordberg A, Ringheim A, Wall A, Forsberg A, et al Unidirectional Influx and Net Accumulation of PIB. Open Neuroimag J. 2008;2: 114–25. 10.2174/1874440000802010114 19526073 PMC2695622

[14] Gjedde A, Aanerud J, Braendgaard H, Rodell AB. Blood-brain transfer of Pittsburgh compound B in humans. Front Aging Neurosci. 2013;5: 1–9. 10.3389/fnagi.2013.00001 24223554 PMC3819578

[15] Rostomian AH, Madison C, Rabinovici GD, Jagust WJ. Early 11C-PIB frames and 18F-FDG PET measures are comparable: a study validated in a cohort of AD and FTLD patients. J Nucl Med. 2011;52: 173–179. 10.2967/jnumed.110.082057 21233181 PMC3166243

[16] Rodriguez-Vieitez E, Carter SF, Chiotis K, Saint-Aubert L, Leuzy A, Scholl M, et al Comparison of Early-Phase 11C-Deuterium-L-Deprenyl and 11C-Pittsburgh Compound B PET for Assessing Brain Perfusion in Alzheimer Disease. J Nucl Med. 2016;57: 1071–1077. 10.2967/jnumed.115.168732 26912447

[17] Forsberg A, Engler H, Blomquist G, Långström B, Nordberg A. The use of PIB-PET as a dual pathological and functional biomarker in AD. Biochim Biophys Acta—Mol Basis Dis. Elsevier B.V.; 2012;1822: 380–385. 10.1016/j.bbadis.2011.11.006 22115832

[18] Tiepolt S, Hesse S, Patt M, Luthardt J, Schroeter ML, Hoffmann KT, et al Early [18F]florbetaben and [11C]PiB PET images are a surrogate biomarker of neuronal injury in Alzheimer’s disease. Eur J Nucl Med Mol Imaging. European Journal of Nuclear Medicine and Molecular Imaging; 2016;43: 1700–1709. 10.1007/s00259-016-3353-1 27026271

[19] Price JC, Klunk WE, Lopresti BJ, Lu X, Hoge JA, Ziolko SK, et al Kinetic Modeling of Amyloid Binding in Humans using PET Imaging and Pittsburgh Compound-B. J Cereb Blood Flow Metab. 2005;25: 1528–1547. 10.1038/sj.jcbfm.9600146 15944649

[20] Wu Y, Carson RE. Noise Reduction in the Simplified Reference Tissue Model for Neuroreceptor Functional Imaging. J Cereb Blood Flow Metab. 2002;22: 1440–1452. 10.1097/01.WCB.0000033967.83623.34 12468889

[21] Yaqub M, Tolboom N, Boellaard R, van Berckel BNM, van Tilburg EW, Luurtsema G, et al Simplified parametric methods for [11C]PIB studies. Neuroimage. 2008;42: 76–86. 10.1016/j.neuroimage.2008.04.251 18541442

[22] Jueptner M, Weiller C. Review: does measurement of regional cerebral blood flow reflect synaptic activity? Implications for PET and fMRI. Neuroimage. 1995;2: 148–56. S1053811985710178 [pii] 9343597 10.1006/nimg.1995.1017

[23] Paulson OB, Hasselbalch SG, Rostrup E, Knudsen GM, Pelligrino D. Cerebral blood flow response to functional activation. J Cereb Blood Flow Metab. Nature Publishing Group; 2010;30: 2–14. 10.1038/jcbfm.2009.188 19738630 PMC2872188

[24] Bélanger M, Allaman I, Magistretti PJJ. Brain Energy Metabolism: Focus on Astrocyte-Neuron Metabolic Cooperation. Cell Metab. 2011;14: 724–738. 10.1016/j.cmet.2011.08.016 22152301

[25] Chen YJ, Rosario BL, Mowrey W, Laymon CM, Lu X, Lopez OL, et al Relative 11C-PiB Delivery as a Proxy of Relative CBF: Quantitative Evaluation Using Single-Session 15O-Water and 11C-PiB PET. J Nucl Med. 2015;56: 1199–1205. 10.2967/jnumed.114.152405 26045309 PMC4730871

[26] McKhann GM, Knopman DS, Chertkow H, Hyman BT, Jack CR, Kawas CH, et al The diagnosis of dementia due to Alzheimer’s disease: Recommendations from the National Institute on Aging-Alzheimer’s Association workgroups on diagnostic guidelines for Alzheimer’s disease. Alzheimer’s Dement. Elsevier Ltd; 2011;7: 263–269. 10.1016/j.jalz.2011.03.005 21514250 PMC3312024

[27] Petersen R, Doody R, Kurz A, Al E. Current concepts in mild cognitive impairment. Arch Neurol. 2001;58: 1985–1992. Available: 10.1001/archneur.58.12.1985 11735772

[28] Ashburner J, Friston KJ. Unified segmentation. Neuroimage. 2005;26: 839–51. 10.1016/j.neuroimage.2005.02.018 15955494

[29] Hammers A, Allom R, Koepp MJ, Free SL, Myers R, Lemieux L, et al Three-dimensional maximum probability atlas of the human brain, with particular reference to the temporal lobe. Hum Brain Mapp. 2003;19: 224–247. 10.1002/hbm.10123 12874777 PMC6871794

[30] Joachim CL, Morris JH, Selkoe DJ. Diffuse senile plaques occur commonly in the cerebellum in Alzheimer’s disease. Am J Pathol. 1989;135: 309–19. Available: http://www.pubmedcentral.nih.gov/articlerender.fcgi?artid=1879919&tool=pmcentrez&rendertype=abstract 2675616 PMC1879919

[31] Yamaguchi H, Hirai S, Morimatsu M, Shoji M, Nakazato Y. Diffuse type of senile plaques in the cerebellum of Alzheimer-type dementia demonstrated by β protein immunostain. Acta Neuropathol. 1989;77: 314–319. 10.1007/BF00687584 2466390

[32] Lammertsma AA, Hume SP. Simplified Reference Tissue Model for PET Receptor Studies. Neuroimage. 1996;4: 153–158. 10.1006/nimg.1996.0066 9345505

[33] Rousset O G; Ma YMEAC, Rousset OG, Ma Y, Evans AC. Correction for partial volume effects in PET: Principle and Validation. J Nucl Med. 1998;39: 904–11. 9591599 9591599

[34] Krouwer JS. Why Bland-Altman plots should use X, not (Y+X)/2 when X is a reference method. Stat Med. 2008;27: 778–80. 10.1002/sim.3086 17907247

[35] R Development Core Team. R: A Language and Environment for Statistical Computing. Vienna; 2017.

[36] Morbelli S, Brugnolo A, Bossert I, Buschiazzo A, Frisoni GB, Galluzzi S, et al Visual Versus semi-quantitative analysis of18F-FDG-PET in Amnestic MCI: An European Alzheimer’s Disease Consortium (EADC) project. J Alzheimer’s Dis. 2015;44: 815–826. 10.3233/JAD-142229 25362041

[37] Lopresti BJ, Klunk WE, Mathis CA, Hoge JA, Ziolko SK, Lu X, et al Simplified Quantification of Pittsburgh Compound B Amyloid Imaging PET Studies: A Comparative Analysis. Time. 2005; 1959–1972. 16330558

[38] Zhou Y, Resnick SM, Ye W, Fan H, Holt DP, Klunk WE, et al Using a reference tissue model with spatial constraint to quantify [11C]Pittsburgh compound B PET for early diagnosis of Alzheimer’s disease. Neuroimage. Elsevier Inc.; 2007;36: 298–312. 10.1016/j.neuroimage.2007.03.004 17449282 PMC2001263

[39] Chen YJ, Rosario BL, Mowrey W, Laymon CM, Lu X, Lopez OL, et al Relative 11C-PiB Delivery as a Proxy of Relative CBF: Quantitative Evaluation Using Single-Session 15O-Water and 11C-PiB PET. J Nucl Med. 2015;56: 1199–205. 10.2967/jnumed.114.152405 26045309 PMC4730871

[40] Rodriguez-Vieitez E, Leuzy A, Chiotis K, Saint-Aubert L, Wall A, Nordberg A. Comparability of [(18)F]THK5317 and [(11)C]PIB blood flow proxy images with [(18)F]FDG positron emission tomography in Alzheimer’s disease. J Cereb Blood Flow Metab. 2017;37: 740–749. 10.1177/0271678X16645593 27107028 PMC5381463

[41] Daerr S, Brendel M, Zach C, Mille E, Schilling D, Zacherl MJ, et al Evaluation of early-phase [18F]-florbetaben PET acquisition in clinical routine cases. NeuroImage Clin. The Author(s); 2017;14: 77–86. 10.1016/j.nicl.2016.10.005 28138429 PMC5257027

[42] Hsiao IT, Huang CC, Hsieh CJ, Wey SP, Kung MP, Yen TC, et al Perfusion-like template and standardized normalization-based brain image analysis using18F-florbetapir (AV-45/Amyvid) PET. Eur J Nucl Med Mol Imaging. 2013;40: 908–920. 10.1007/s00259-013-2350-x 23412134

[43] Hsiao IT, Huang CC, Hsieh CJ, Hsu WC, Wey SP, Yen TC, et al Correlation of early-phase18F-florbetapir (AV-45/Amyvid) PET images to FDG images: Preliminary studies. Eur J Nucl Med Mol Imaging. 2012;39: 613–620. 10.1007/s00259-011-2051-2 22270508

[44] Greve DN, Salat DH, Bowen SL, Izquierdo-Garcia D, Schultz AP, Catana C, et al Different partial volume correction methods lead to different conclusions: An18F-FDG-PET study of aging. Neuroimage. Elsevier Inc.; 2016;132: 334–343. 10.1016/j.neuroimage.2016.02.042 26915497 PMC4851886

[45] Greve DN, Svarer C, Fisher PM, Feng L, Hansen AE, Baare W, et al Cortical surface-based analysis reduces bias and variance in kinetic modeling of brain PET data. Neuroimage. Elsevier Inc.; 2014;92: 225–236. 10.1016/j.neuroimage.2013.12.021 24361666 PMC4008670

[46] Gur RC, Ragland JD, Reivich M, Greenberg JH, Alavi A, Gur RE. Regional differences in the coupling between resting cerebral blood flow and metabolism may indicate action preparedness as a default state. Cereb Cortex. 2009;19: 375–382. 10.1093/cercor/bhn087 18534991 PMC2638785

[47] Iadecola C. Neurovascular regulation in the normal brain and in Alzheimer’s disease. Nat Rev Neurosci. 2004;5: 347–360. 10.1038/nrn1387 15100718

