# Correction: Relative cerebral flow from dynamic PIB scans as an alternative for FDG scans in Alzheimer's disease PET studies

**DOI:** 10.1371/journal.pone.0214187

**Published:** 2019-03-18

**Authors:** Débora E. Peretti, David Vállez García, Fransje E. Reesink, Tim van der Goot, Peter P. De Deyn, Bauke M. de Jong, Rudi A. J. O. Dierckx, Ronald Boellaard

There is an error in the fourth sentence of the second paragraph of the “Image processing” subsection of the Materials and Methods. The correct sentence is: Then, the *k’*_*2*_ parameter was defined as the median value from all voxels that have a *BP*_nd_ value higher than 0.05.

There is an error in the third sentence of the third paragraph of the “Group differences” subsection of the Results. The correct sentence is: The region that presented the highest values was, for both groups, the Putamen (1.14 ± 0.07 for the PIB+ group and 1.13 ± 0.05 for the PIB- group).

There is an error in the DOI of reference 14. The correct reference is: Gjedde A, Aanerud J, Braendgaard H, Rodell AB. Blood-brain transfer of Pittsburgh compound B in humans. Front Aging Neurosci. 2013;5: 1–9. https://doi.org/10.3389/fnagi.2013.00070.

The images for Figs 4–6 are incorrectly switched. The image that appears as Fig 4 should be Fig 6; the image that appears as Fig 5 should be Fig 4, and the image that appears as Fig 6 should be Fig 5. The figure captions appear in the correct order. Please see the correct figures and their respective captions here.

**Fig 4:**
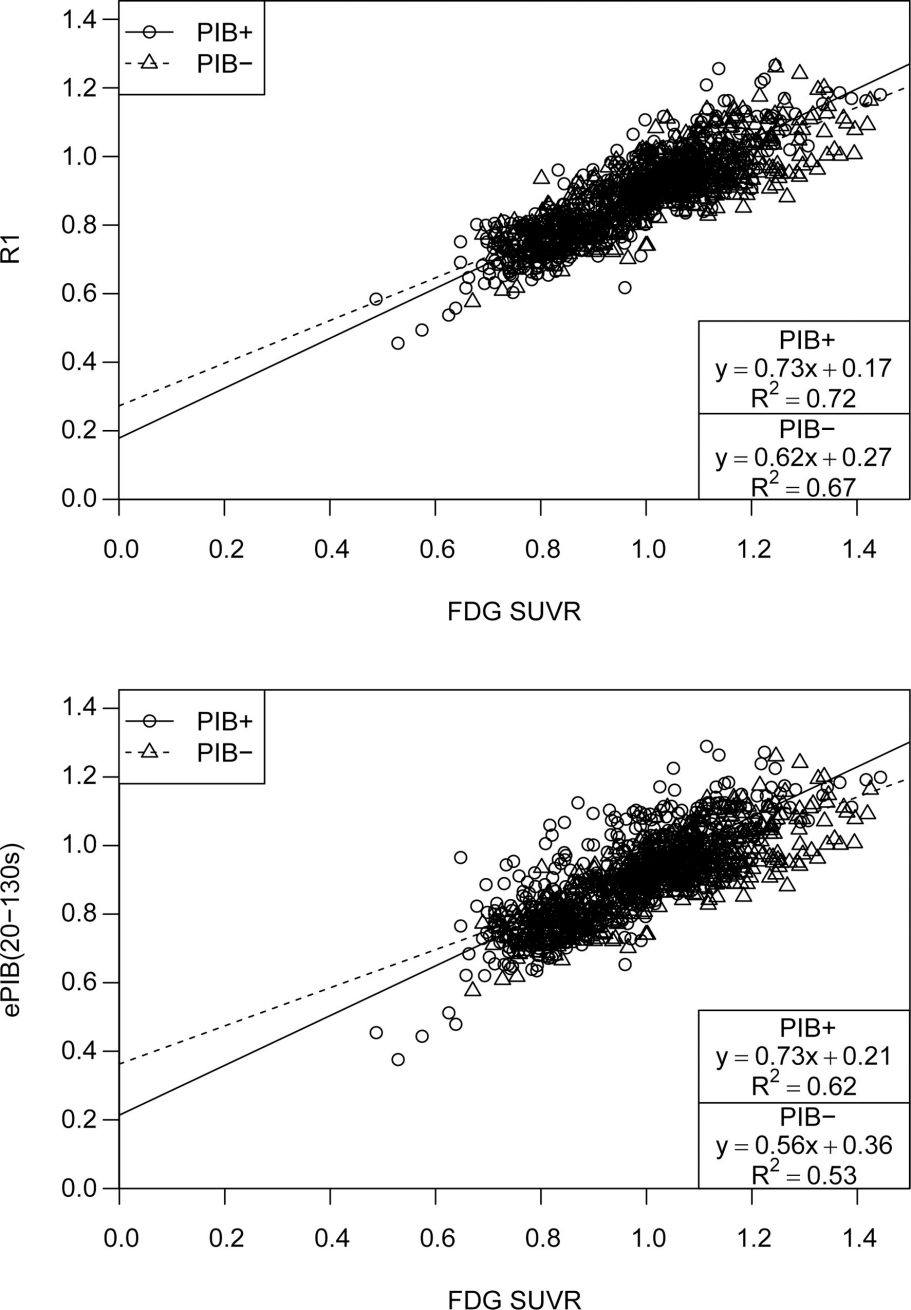
Linear regression analysis for R1 and ePIB(20-130s) estimates. Scatter plot showing regional CBF estimates from R_1_ parametric images (top) and ePIB(20-130s; bottom) (y-axis), and normalized FDG FDG uptake (x-axis). Data are arranged according to patient group: circles represent PIB+ group, and triangles PIB-. Lines resulting from the linear regression applied to the data are also shown: a full line for the PIB+ group, and a dashed one for PIB-. Results of the linear regression are given in boxes at the bottom right corner.

**Fig 5:**
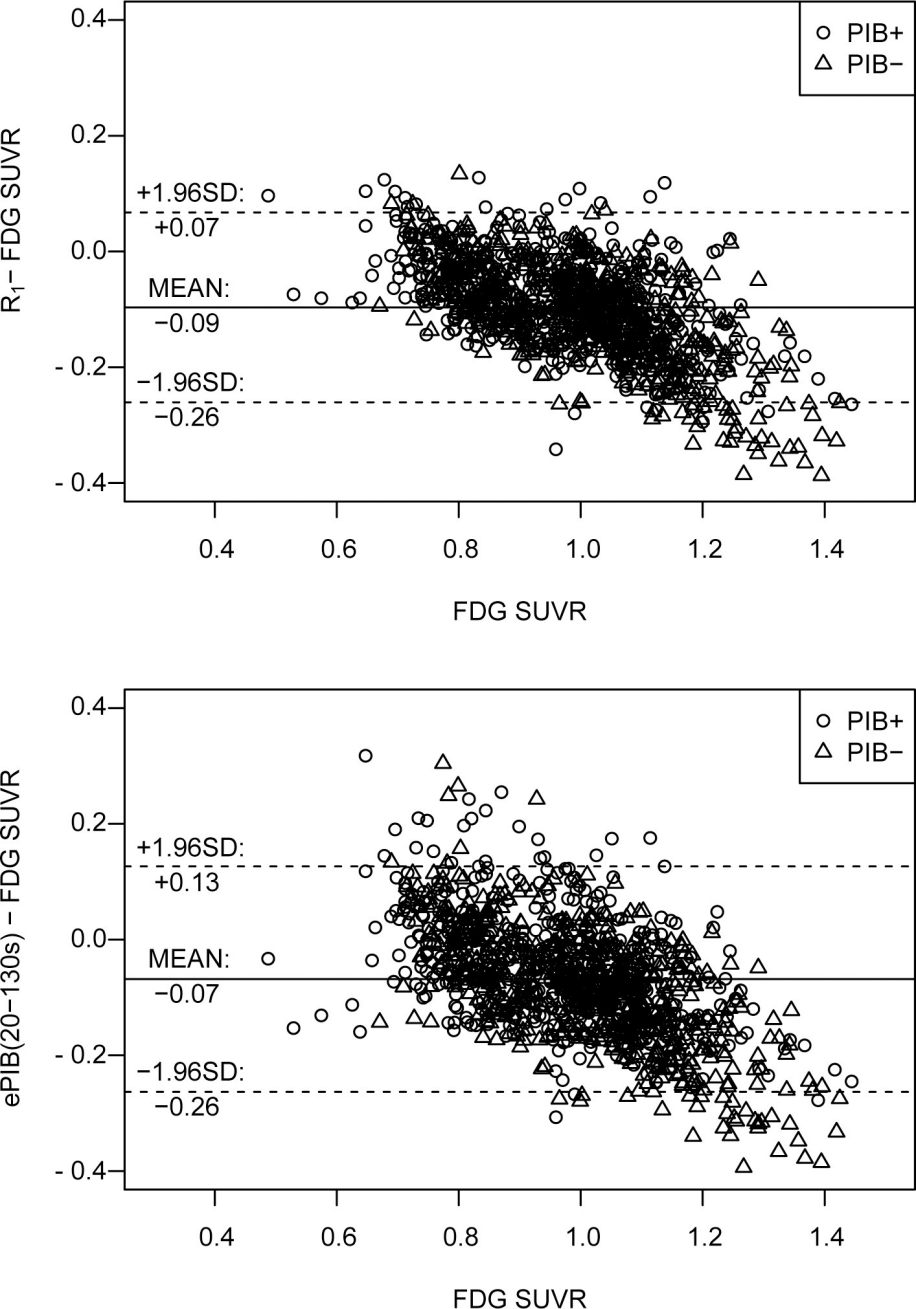
Bland-Altman plot. Bland-Altman plot showing the difference between the values of rCBF assessed by different methods (by *R*_1_, on the top row, and by ePIB(20 to 130 seconds), on the bottom, estimations and from the normalized FDG uptake). Circles represent data from the PIB+ group, while triangles represent PIB-. The full line is at the mean difference value for all data (not classified in groups), and the dashed lines delimit the 95% agreement interval (at mean ± 1.96 × standard deviation).

**Fig 6:**
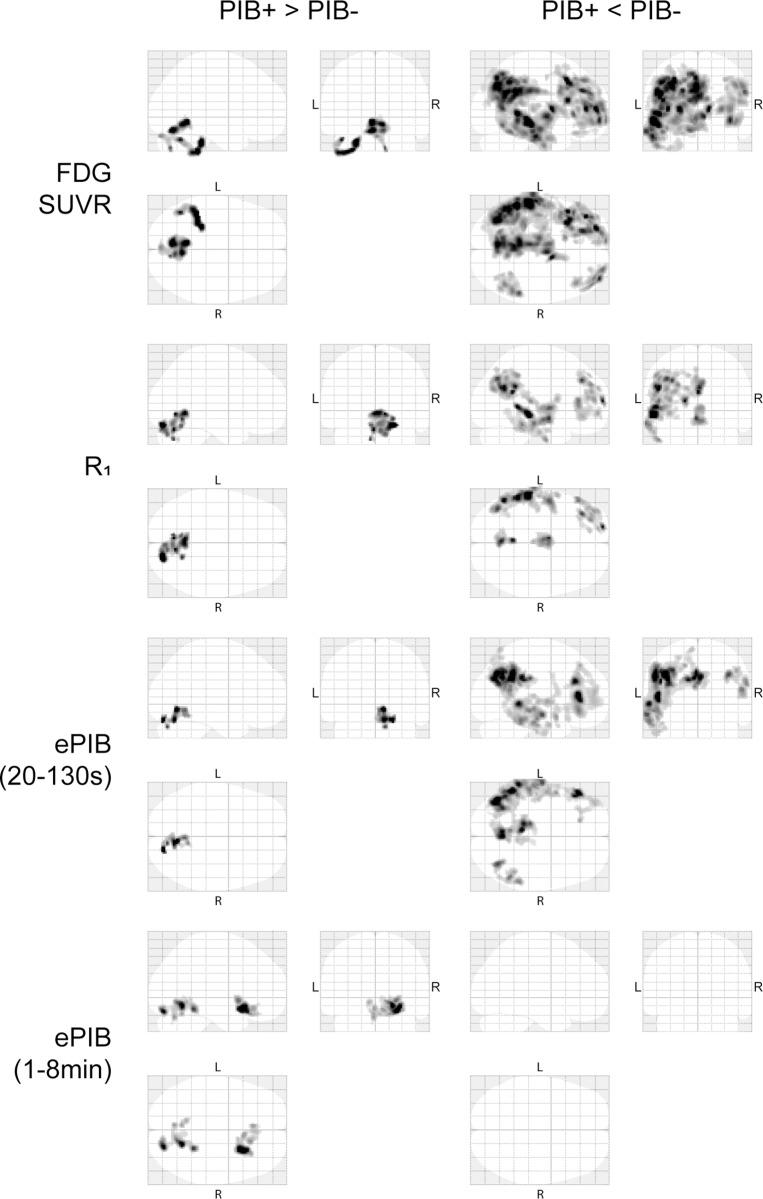
SPM analysis. Maximum Intensity Projections derived from the voxel based analysis. First row contains the images from FDG SUVR, second row shows *R*_1_, third, ePIB(20 to 130 seconds), and fourth, ePIB(1 to 8 minutes). On the left, statistically significant regions where PIB+ group shows higher rCBF than the PIB- group, and, on the right, statistically significant regions where the PIB- group showed higher flow than the PIB+ group.
