# Supplementary figures and images for: Relative cerebral flow from dynamic PIB scans as an alternative for FDG scans in Alzheimer’s disease PET studies
Source: PLoS One. 2019 Jan 17;14(1):e0211000. doi: 10.1371/journal.pone.0211000 (PMC6336325; doi:10.1371/journal.pone.0211000)

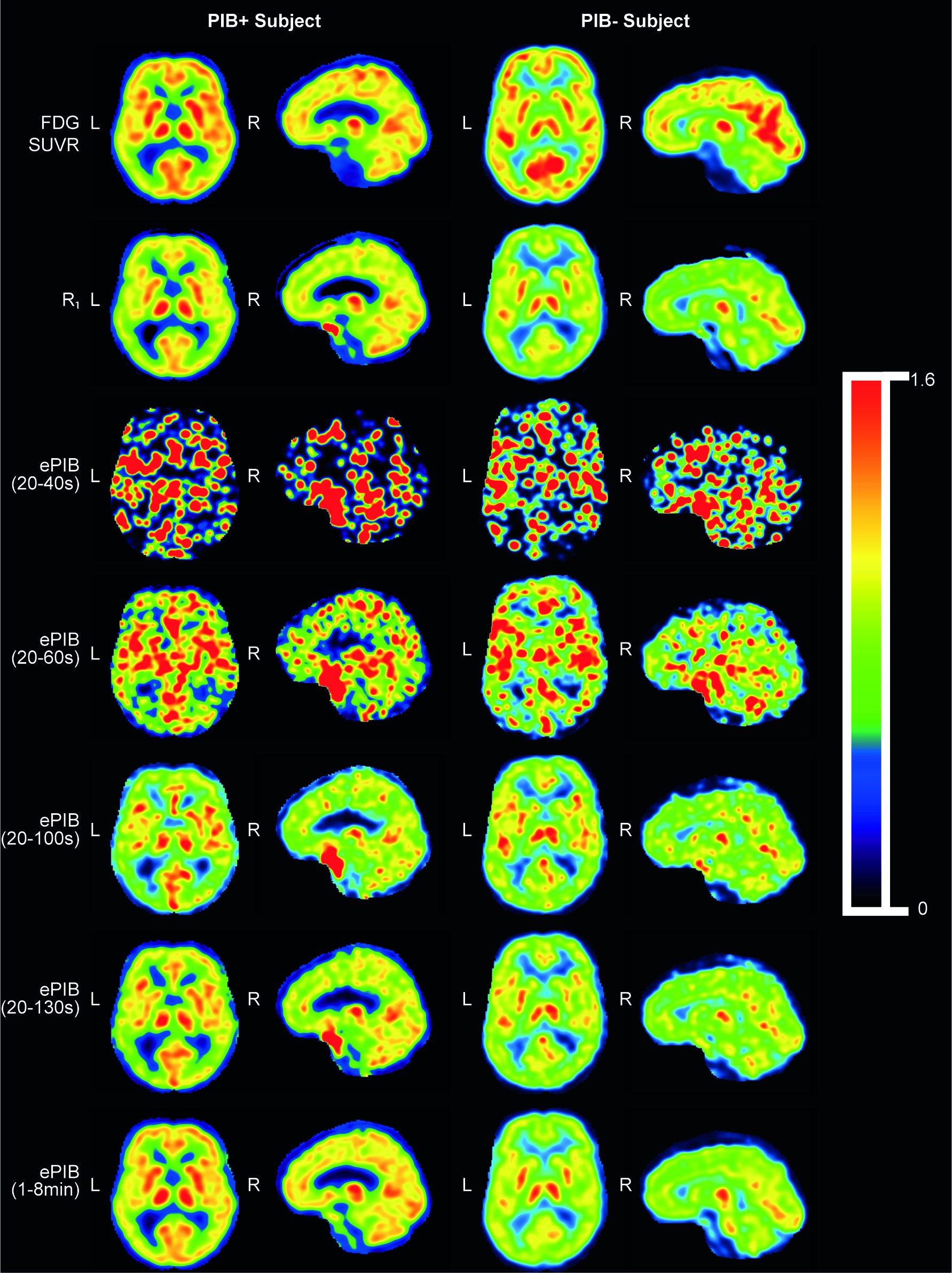

Supplement: S1 Fig — Representative images of normalized FDG uptake images (first row), parametric images of PiB rCBF (R1; second row), and all time weighted average of early PIB frames (20 to 40 seconds on the third row, 20 to 60 seconds on the fourth, 20 to 100 seconds on the fifth, 20 to 130 seconds on the sixth, and 1 to 8 minutes on the seventh row) of a PIB+ patient (left), and a PIB- subject (right). Shown are corresponding transaxial, and sagittal slices of the brain. All colour scales were adjusted to the same range. (TIF) [file pone.0211000.s001.tif]

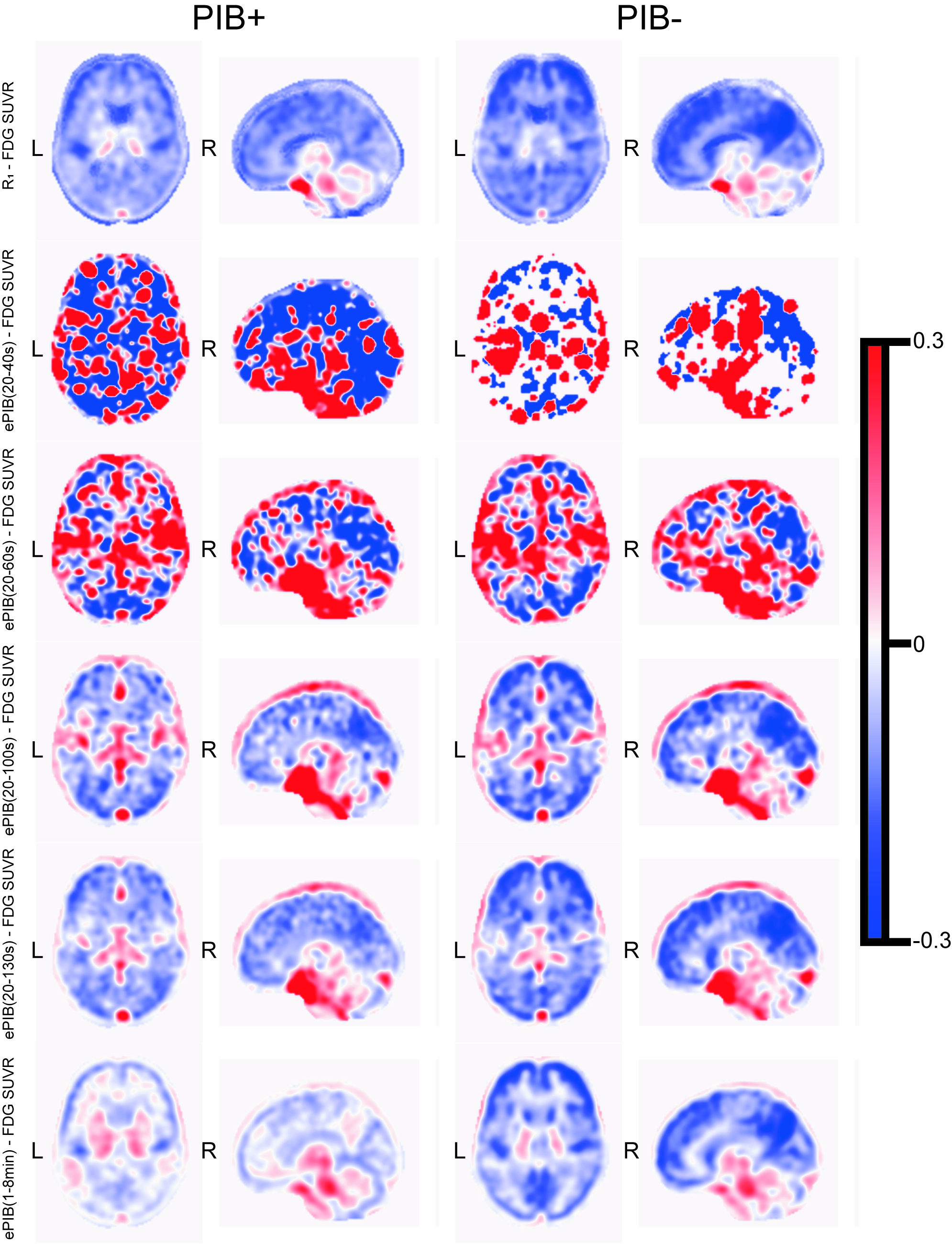

Supplement: S2 Fig — Mean difference images per groups comparing normalized FDG uptake and R1 parametric maps (R1 –SUVR; first row), and for all ePIB time intervals: 20 to 40 seconds (second row), 20 to 60 seconds (third row), 20 to 100 seconds (fourth row), 20 to 130 seconds (fifth row), and 1 to 8 minutes (sixth row). On the left, the mean difference image for the PIB+ group can be seen, and on the right, the PIB-. The closer the rCBF and SUVR estimates, the more white the voxel appears. Negative values correspond to voxels where the SUVR voxel presented a higher value than the R1 or ePIB. (TIF) [file pone.0211000.s002.tif]

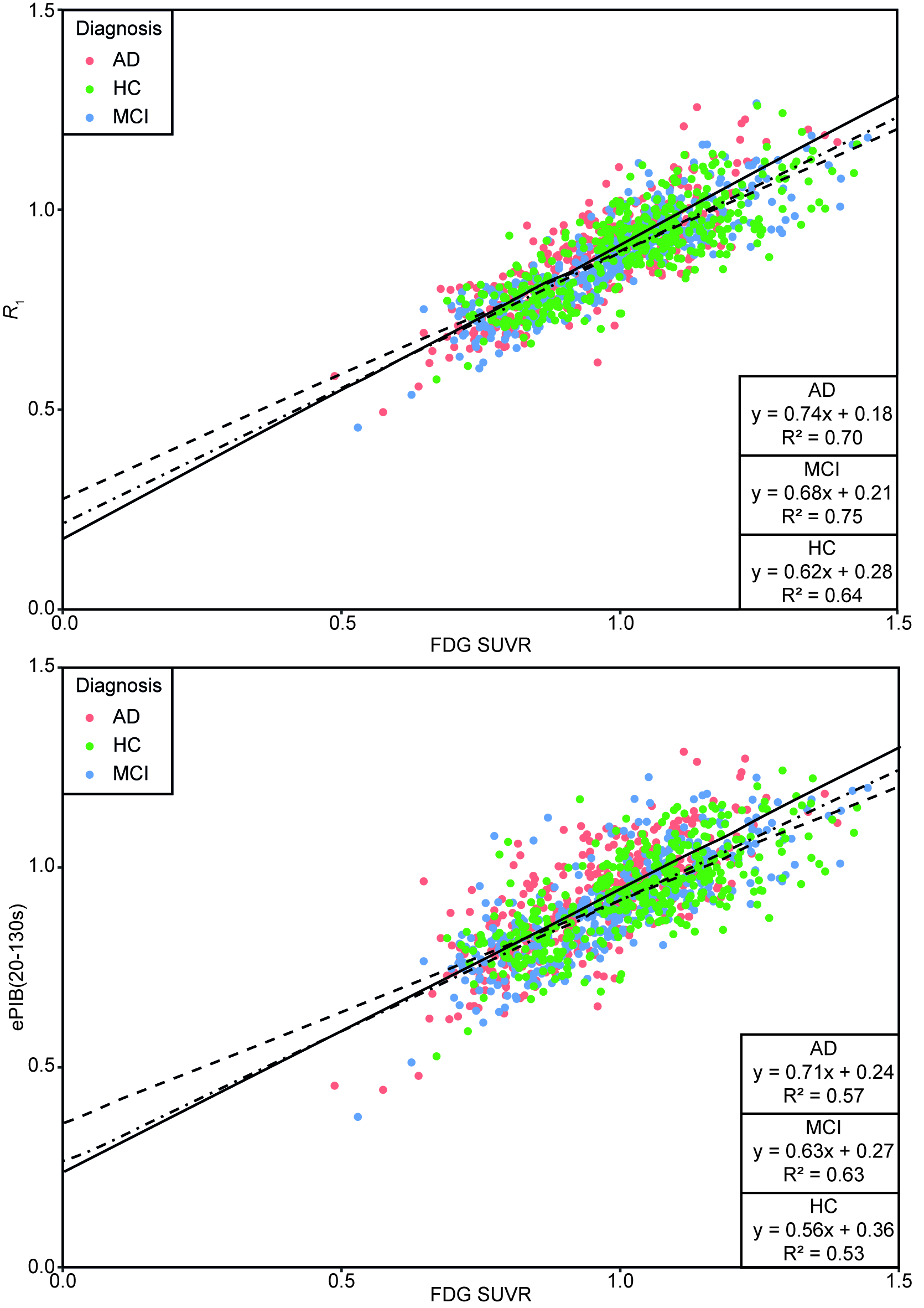

Supplement: S3 Fig — Scatter plot showing regional CBF estimates from R1 parametric images (top) and ePIB(20-130s; bottom) (y-axis), and normalized FDG uptake (x-axis). Data are arranged according to subject diagnosis: red points represent AD patients, green points represent the HC subjects, and blue points represent MCI participants. Lines resulting from the linear regression applied to the data are also shown: a full line for the AD group, a dashed one for HC subjects, and a dot and dash line for the MCI participants. Results of the linear regression are given in boxes at the bottom right corner. (TIF) [file pone.0211000.s003.tif]

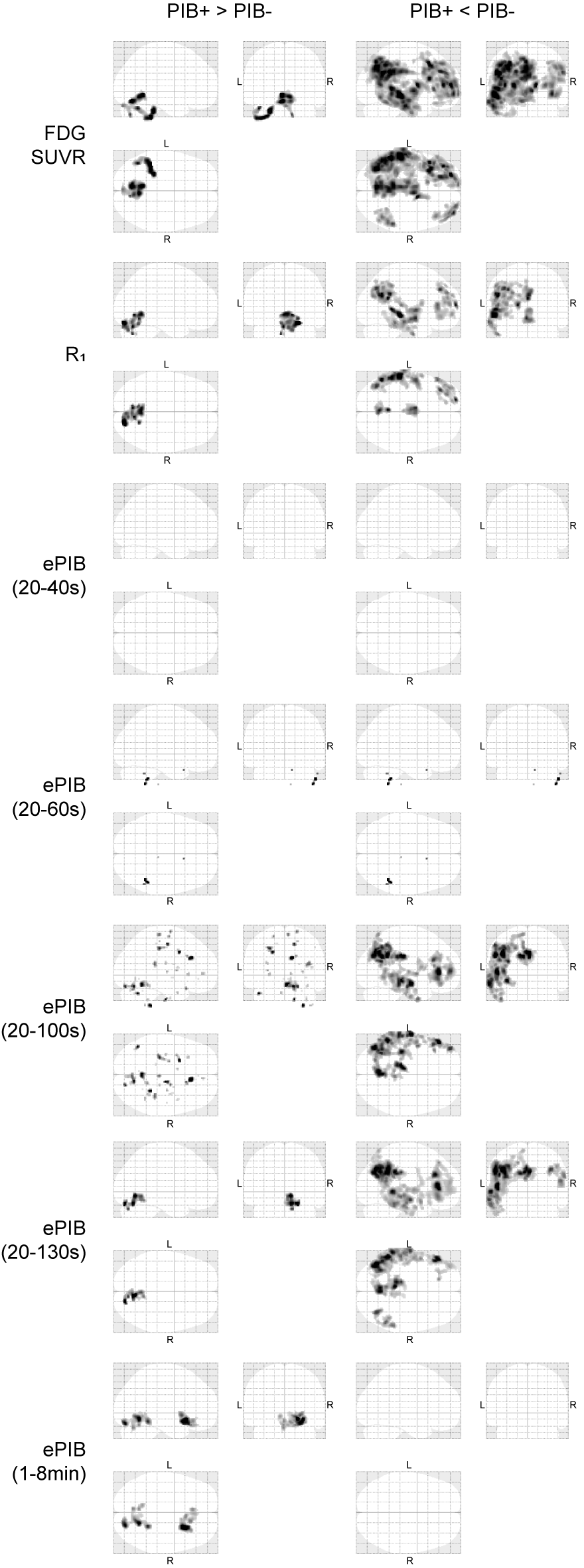

Supplement: S4 Fig — Maximum Intensity Projections derived from the voxel based analysis. The rows contain, in order from top to bottom, FDG SUVR, R1, ePIB(20-40s), ePIB(20-60s), ePIB(20-100s), ePIB(20-130s), and ePIB(1-8min). On the left, statistically significant regions where PIB+ group shows higher rCBF than the PIB- group, and, on the right, statistically significant regions where the PIB- group showed higher flow than the PIB+ group. (TIF) [file pone.0211000.s004.tif]
