# Supplementary material for: Relative cerebral flow from dynamic PIB scans as an alternative for FDG scans in Alzheimer’s disease PET studies
Source: PLoS One. 2019 Jan 17;14(1):e0211000. doi: 10.1371/journal.pone.0211000 (PMC6336325; doi:10.1371/journal.pone.0211000)
Supplement: S1 Table — List of all regions of interest included and their separation in this study. (DOCX) [file pone.0211000.s005.docx]

| **Brain Areas** | **Name of Region** |
| --- | --- |
| Frontal Lobe | Superior frontal gyrus |
|  | Middle frontal gyrus |
|  | Inferior frontal gyrus |
|  | Precentral gyrus |
|  | Straight gyrus |
|  | Anterior orbital gyrus |
|  | Lateral orbital gyrus |
|  | Medial orbital gyrus |
|  | Posterior orbital gyrus |
|  | Subcallosal area |
|  | Subgenual frontal cortex |
|  | Pre-subgenual frontal cortex |
| Occipital Lobe | Cuneus |
|  | Lingual gyrus |
|  | Lateral remainder of occipital lobe |
| Temporal Lobe | Hippocampus |
|  | Amygdala |
|  | Anterior temporal lobe lateral part |
|  | Anterior temporal lobe medial part |
|  | Parahippocampal and ambient gyri |
|  | Superior temporal gyrus anterior part |
|  | Superior temporal gyrus posterior part |
|  | Middle and inferior temporal gyrus |
|  | Fusiform gyrus |
|  | Posterior temporal lobe |
| Parietal Lobe | Postcentral gyrus |
|  | Superior parietal gyrus |
|  | Inferiolateral remainder of parietal lobe |
| Central Structures | Caudate nucleus |
|  | Nucleus accumbens |
|  | Putamen |
|  | Thalamus |
|  | Pallidum |
|  | Substantia nigra |
| Insula and Cingulate gyri | Insula |
|  | Cingulate gyrus anterior part |
|  | Cingulate gyrus posterior part |
| Posterior Fossa | Brainstem |
|  | Cerebellum |
| White Matter | White matter |
