# Supplementary material for: Relative cerebral flow from dynamic PIB scans as an alternative for FDG scans in Alzheimer’s disease PET studies
Source: PLoS One. 2019 Jan 17;14(1):e0211000. doi: 10.1371/journal.pone.0211000 (PMC6336325; doi:10.1371/journal.pone.0211000)
Supplement: S3 Table — R1 values (expressed as mean ± standard deviation) for each region per subject group, and uncorrected and corrected for false discovery rate p-values from the t-test. (DOCX) [file pone.0211000.s007.docx]

| **Region** | **PIB+** | **PIB-** | **p-value^unc^** | **p-value^FDR^** |
| --- | --- | --- | --- | --- |
| Superior frontal gyrus | 0.98 ± 0.06 | 0.92 ± 0.04 | 0.14 | 0.48 |
| Middle frontal gyrus | 0.93 ± 0.06 | 0.98 ± 0.05 | 0.02 * | 0.08 |
| Inferior frontal gyrus | 0.94 ± 0.06 | 0.99 ± 0.04 | 0.01 * | 0.08 |
| Precentral gyrus | 0.91 ± 0.06 | 0.93 ± 0.03 | 0.40 | 0.60 |
| Straight gyrus | 0.92 ± 0.07 | 0.94 ± 0.05 | 0.38 | 0.60 |
| Anterior orbital gyrus | 0.92 ± 0.09 | 0.95 ± 0.05 | 0.33 | 0.60 |
| Lateral orbital gyrus | 0.89 ± 0.10 | 0.93 ± 0.05 | 0.21 | 0.54 |
| Medial orbital gyrus | 0.89 ± 0.08 | 0.91 ± 0.04 | 0.40 | 0.60 |
| Posterior orbital gyrus | 0.93 ± 0.07 | 0.93 ± 0.04 | 0.88 | 0.91 |
| Subcallosal area | 0.78 ± 0.09 | 0.81 ± 0.06 | 0.25 | 0.57 |
| Subgenual frontal cortex | 0.84 ± 0.05 | 0.84 ± 0.06 | 0.85 | 0.91 |
| Pre-subgenual frontal cortex | 0.97 ± 0.06 | 0.97 ± 0.08 | 0.89 | 0.91 |
| Cuneus | 1.06 ± 0.08 | 1.10 ± 0.06 | 0.31 | 0.60 |
| Lingual gyrus | 1.08 ± 0.07 | 1.07 ± 0.05 | 0.52 | 0.65 |
| Lateral remainder of occipital lobe | 0.92 ± 0.08 | 0.93 ± 0.04 | 0.56 | 0.68 |
| Hippocampus | 0.76 ± 0.05 | 0.79 ± 0.04 | 0.05 | 0.20 |
| Amygdala | 0.74 ± 0.05 | 0.75 ± 0.02 | 0.19 | 0.54 |
| Anterior temporal lobe lateral part | 0.76 ± 0.04 | 0.80 ± 0.04 | 0.01 * | 0.08 |
| Anterior temporal lobe medial part | 0.71 ± 0.04 | 0.72 ± 0.02 | 0.37 | 0.60 |
| Parahippocampal and ambient gyri | 0.73 ± 0.05 | 0.73 ± 0.04 | 0.73 | 0.82 |
| Superior temporal gyrus anterior part | 0.76 ± 0.05 | 0.78 ± 0.06 | 0.21 | 0.54 |
| Superior temporal gyrus posterior part | 0.89 ± 0.06 | 0.96 ± 0.07 | < 0.01 * | 0.06 |
| Middle and inferior temporal gyrus | 0.83 ± 0.06 | 0.90 ± 0.03 | < 0.01 * | 0.02 * |
| Fusiform gyrus | 0.77 ± 0.06 | 0.78 ± 0.03 | 0.45 | 0.62 |
| Posterior temporal lobe | 0.89 ± 0.05 | 0.93 ± 0.03 | 0.01 * | 0.07 |
| Postcentral gyrus | 0.87 ± 0.06 | 0.89 ± 0.05 | 0.43 | 0.61 |
| Superior parietal gyrus | 0.90 ± 0.08 | 0.95 ± 0.05 | 0.04 * | 0.14 |
| Inferiolateral remainder of parietal lobe | 0.86 ± 0.07 | 0.94 ± 0.05 | < 0.01 * | 0.02 * |
| Caudate nucleus | 0.66 ± 0.14 | 0.71 ± 0.07 | 0.25 | 0.57 |
| Nucleus accumbens | 0.92 ± 0.07 | 0.93 ± 0.07 | 0.69 | 0.79 |
| Putamen | 1.14 ± 0.07 | 1.13 ± 0.05 | 0.52 | 0.65 |
| Thalamus | 0.98 ± 0.09 | 1.00 ± 0.06 | 0.39 | 0.60 |
| Pallidum | 0.94 ± 0.08 | 0.94 ± 0.07 | 0.96 | 0.96 |
| Substantia nigra | 0.74 ± 0.05 | 0.80 ± 0.06 | < 0.01 * | 0.04 * |
| Insula | 0.92 ± 0.06 | 0.93 ± 0.05 | 0.48 | 0.64 |
| Cingulate gyrus anterior part | 0.91 ± 0.08 | 0.94 ± 0.06 | 0.33 | 0.60 |
| Cingulate gyrus posterior part | 0.99 ± 0.08 | 1.07 ± 0.05 | < 0.01 * | 0.04 * |
| Brainstem | 0.77 ± 0.03 | 0.78 ± 0.02 | 0.20 | 0.54 |
| Cerebellum | 1.01 ± 0.006 | 1.00 ± 0.004 | 0.32 | 0.60 |
| White matter | 0.73 ± 0.05 | 0.74 ± 0.03 | 0.68 | 0.79 |

* Statistically significant values.
