# Supplementary material for: Relative cerebral flow from dynamic PIB scans as an alternative for FDG scans in Alzheimer’s disease PET studies
Source: PLoS One. 2019 Jan 17;14(1):e0211000. doi: 10.1371/journal.pone.0211000 (PMC6336325; doi:10.1371/journal.pone.0211000)
Supplement: S4 Table — ePIB(20-40s) values (expressed as mean ± standard deviation) for each region per subject group, and uncorrected and corrected for false discovery rate p-values from the t-test. (DOCX) [file pone.0211000.s008.docx]

| **Region** | **PIB+** | **PIB-** | **p-value^unc^** | **p-value^FDR^** |
| --- | --- | --- | --- | --- |
| Superior frontal gyrus | 0.63 ± 0.47 | 0.82 ± 0.33 | 0.22 | 0.66 |
| Middle frontal gyrus | 1.07 ± 1.22 | 0.61 ± 0.34 | 0.18 | 0.66 |
| Inferior frontal gyrus | 0.96 ± 0.63 | 0.87 ± 0.35 | 0.62 | 0.92 |
| Precentral gyrus | 0.66 ± 0.45 | 0.70 ± 0.34 | 0.80 | 0.92 |
| Straight gyrus | 1.31 ± 0.88 | 0.89 ± 0.56 | 0.13 | 0.66 |
| Anterior orbital gyrus | 0.63 ± 0.41 | 0.61 ± 0.34 | 0.88 | 0.93 |
| Lateral orbital gyrus | 0.72 ± 0.48 | 1.06 ± 0.62 | 0.11 | 0.66 |
| Medial orbital gyrus | 1.56 ± 2.13 | 0.79 ± 0.37 | 0.17 | 0.66 |
| Posterior orbital gyrus | 2.00 ± 1.91 | 1.31 ± 0.67 | 0.20 | 0.66 |
| Subcallosal area | 1.19 ± 0.91 | 1.04 ± 1.52 | 0.75 | 0.92 |
| Subgenual frontal cortex | 2.60 ± 4.99 | 1.06 ± 1.40 | 0.26 | 0.66 |
| Pre-subgenual frontal cortex | 1.23 ± 0.84 | 1.31 ± 1.16 | 0.84 | 0.92 |
| Cuneus | 1.78 ± 2.66 | 0.76 ± 0.46 | 0.16 | 0.66 |
| Lingual gyrus | 2.09 ± 4.51 | 0.84 ± 0.52 | 0.29 | 0.66 |
| Lateral remainder of occipital lobe | 0.89 ± 0.65 | 0.77 ± 0.37 | 0.54 | 0.87 |
| Hippocampus | 1.31 ± 1.35 | 1.01 ± 0.58 | 0.44 | 0.76 |
| Amygdala | 1.99 ± 1.62 | 1.31 ± 0.81 | 0.16 | 0.66 |
| Anterior temporal lobe lateral part | 2.19 ± 4.24 | 1.01 ± 0.87 | 0.30 | 0.66 |
| Anterior temporal lobe medial part | 1.50 ± 1.49 | 1.00 ± 0.52 | 0.22 | 0.66 |
| Parahippocampal and ambient gyri | 1.50 ± 0.90 | 1.05 ± 0.60 | 0.11 | 0.66 |
| Superior temporal gyrus anterior part | 1.53 ± 0.92 | 1.21 ± 0.65 | 0.27 | 0.66 |
| Superior temporal gyrus posterior part | 1.96 ± 2.46 | 1.15 ± 0.59 | 0.23 | 0.66 |
| Middle and inferior temporal gyrus | 1.31 ± 1.12 | 0.97 ± 0.48 | 0.29 | 0.66 |
| Fusiform gyrus | 1.11 ± 0.50 | 1.54 ± 0.72 | 0.83 | 0.92 |
| Posterior temporal lobe | 1.94 ± 3.15 | 0.98 ± 0.45 | 0.25 | 0.66 |
| Postcentral gyrus | 1.29 ± 2.22 | 0.78 ± 0.43 | 0.39 | 0.73 |
| Superior parietal gyrus | 0.59 ± 0.34 | 0.61 ± 0.31 | 0.85 | 0.92 |
| Inferiolateral remainder of parietal lobe | 0.81 ± 0.55 | 0.67 ± 0.33 | 0.40 | 0.73 |
| Caudate nucleus | 0.71 ± 0.55 | 0.68 ± 0.39 | 0.86 | 0.92 |
| Nucleus accumbens | 1.08 ± 0.89 | 1.18 ± 1.58 | 0.83 | 0.92 |
| Putamen | 1.51 ± 1.56 | 1.31 ± 0.49 | 0.65 | 0.92 |
| Thalamus | 0.79 ± 0.55 | 0.77 ± 0.46 | 0.92 | 0.94 |
| Pallidum | 1.15 ± 1.93 | 0.89 ± 0.62 | 0.64 | 0.92 |
| Substantia nigra | 2.73 ± 7.37 | 1.06 ± 0.88 | 0.39 | 0.73 |
| Insula | 1.84 ± 2.67 | 1.38 ± 0.65 | 0.52 | 0.87 |
| Cingulate gyrus anterior part | 0.83 ± 0.64 | 0.78 ± 0.40 | 0.81 | 0.92 |
| Cingulate gyrus posterior part | 0.86 ± 0.75 | 0.85 ± 0.64 | 0.99 | 0.99 |
| Brainstem | 1.15 ± 0.54 | 1.04 ± 0.80 | 0.68 | 0.92 |
| Cerebellum | 1.00 ± 0.00 | 1.00 ± 0.00 | 0.21 | 0.66 |
| White matter | 0.99 ± 0.86 | 0.78 ± 0.28 | 0.38 | 0.73 |

* Statistically significant values.
