# Supplementary material for: Relative cerebral flow from dynamic PIB scans as an alternative for FDG scans in Alzheimer’s disease PET studies
Source: PLoS One. 2019 Jan 17;14(1):e0211000. doi: 10.1371/journal.pone.0211000 (PMC6336325; doi:10.1371/journal.pone.0211000)
Supplement: S5 Table — ePIB(20-60s) values (expressed as mean ± standard deviation) for each region per subject group, and uncorrected and corrected for false discovery rate p-values from the t-test. (DOCX) [file pone.0211000.s009.docx]

| **Region** | **PIB+** | **PIB-** | **p-value^unc^** | **p-value^FDR^** |
| --- | --- | --- | --- | --- |
| Superior frontal gyrus | 0.99 ± 0.26 | 1.05 ± 0.20 | 0.46 | 0.90 |
| Middle frontal gyrus | 0.91 ± 0.21 | 0.98 ± 0.17 | 0.35 | 0.82 |
| Inferior frontal gyrus | 1.06 ± 0.21 | 1.17 ± 0.22 | 0.17 | 0.71 |
| Precentral gyrus | 1.04 ± 0.31 | 0.99 ± 0.16 | 0.56 | 0.93 |
| Straight gyrus | 1.40 ± 0.44 | 1.40 ± 0.32 | 0.96 | 0.96 |
| Anterior orbital gyrus | 0.96 ± 0.30 | 1.00 ± 0.24 | 0.67 | 0.93 |
| Lateral orbital gyrus | 0.92 ± 0.16 | 1.03 ± 0.23 | 0.16 | 0.71 |
| Medial orbital gyrus | 1.16 ± 0.51 | 1.08 ± 0.20 | 0.58 | 0.93 |
| Posterior orbital gyrus | 1.40 ± 0.46 | 1.37 ± 0.37 | 0.82 | 0.93 |
| Subcallosal area | 1.39 ± 0.65 | 1.62 ± 0.71 | 0.35 | 0.82 |
| Subgenual frontal cortex | 1.39 ± 0.52 | 1.42 ± 0.29 | 0.89 | 0.95 |
| Pre-subgenual frontal cortex | 1.14 ± 0.32 | 1.43 ± 0.43 | 0.04 | 0.52 |
| Cuneus | 1.10 ± 0.25 | 1.13 ± 0.15 | 0.66 | 0.93 |
| Lingual gyrus | 1.18 ± 0.26 | 1.15 ± 0.15 | 0.71 | 0.93 |
| Lateral remainder of occipital lobe | 0.84 ± 0.15 | 0.93 ± 0.16 | 0.14 | 0.71 |
| Hippocampus | 1.05 ± 0.29 | 1.23 ± 0.40 | 0.17 | 0.71 |
| Amygdala | 1.13 ± 0.27 | 1.27 ± 0.39 | 0.26 | 0.82 |
| Anterior temporal lobe lateral part | 0.84 ± 0.21 | 0.88 ± 0.10 | 0.59 | 0.93 |
| Anterior temporal lobe medial part | 0.99 ± 0.26 | 1.02 ± 0.18 | 0.70 | 0.93 |
| Parahippocampal and ambient gyri | 1.26 ± 0.48 | 1.28 ± 0.27 | 0.91 | 0.95 |
| Superior temporal gyrus anterior part | 1.05 ± 0.21 | 1.13 ± 0.28 | 0.37 | 0.82 |
| Superior temporal gyrus posterior part | 1.17 ± 0.29 | 1.27 ± 0.28 | 0.34 | 0.82 |
| Middle and inferior temporal gyrus | 0.83 ± 0.11 | 1.00 ± 0.29 | 0.44 | 0.52 |
| Fusiform gyrus | 0.95 ± 0.27 | 1.00 ± 0.24 | 0.55 | 0.93 |
| Posterior temporal lobe | 0.95 ± 0.21 | 1.04 ± 0.24 | 0.32 | 0.82 |
| Postcentral gyrus | 1.02 ± 0.21 | 1.00 ± 0.22 | 0.84 | 0.93 |
| Superior parietal gyrus | 0.88 ± 0.18 | 0.98 ± 0.19 | 0.15 | 0.71 |
| Inferiolateral remainder of parietal lobe | 0.93 ± 0.25 | 1.02 ± 0.15 | 0.21 | 0.77 |
| Caudate nucleus | 0.64 ± 0.19 | 0.82 ± 0.28 | 0.05* | 0.52 |
| Nucleus accumbens | 1.34 ± 0.48 | 1.47 ± 0.54 | 0.47 | 0.90 |
| Putamen | 1.40 ± 0.58 | 1.27 ± 0.30 | 0.43 | 0.90 |
| Thalamus | 1.05 ± 0.43 | 1.06 ± 0.18 | 0.93 | 0.96 |
| Pallidum | 1.38 ± 1.28 | 1.05 ± 0.26 | 0.34 | 0.82 |
| Substantia nigra | 1.37 ± 0.83 | 1.05 ± 0.27 | 0.18 | 0.71 |
| Insula | 1.31 ± 0.40 | 1.36 ± 0.42 | 0.76 | 0.93 |
| Cingulate gyrus anterior part | 1.26 ± 0.38 | 1.29 ± 0.32 | 0.77 | 0.93 |
| Cingulate gyrus posterior part | 0.94 ± 0.20 | 1.23 ± 0.29 | <0.01* | 0.11 |
| Brainstem | 1.03 ± 0.25 | 1.04 ± 0.17 | 0.84 | 0.93 |
| Cerebellum | 1.00 ± 0.00 | 1.00 ± 0.00 | 0.77 | 0.93 |
| White matter | 0.81 ± 0.23 | 0.84 ± 0.16 | 0.69 | 0.93 |

* Statistically significant values.
