# Supplementary material for: Relative cerebral flow from dynamic PIB scans as an alternative for FDG scans in Alzheimer’s disease PET studies
Source: PLoS One. 2019 Jan 17;14(1):e0211000. doi: 10.1371/journal.pone.0211000 (PMC6336325; doi:10.1371/journal.pone.0211000)
Supplement: S7 Table — ePIB(20-130s) values (expressed as mean ± standard deviation) for each region per subject group, and uncorrected and corrected for false discovery rate p-values from the t-test. (DOCX) [file pone.0211000.s011.docx]

| **Region** | **PIB+** | **PIB-** | **p-value^unc^** | **p-value^FDR^** |
| --- | --- | --- | --- | --- |
| Superior frontal gyrus | 0.93 ± 0.07 | 0.93 ± 0.04 | 0.88 | 0.99 |
| Middle frontal gyrus | 0.94 ± 0.07 | 0.98 ± 0.05 | 0.06 | 0.33 |
| Inferior frontal gyrus | 0.98 ± 0.07 | 1.02 ± 0.04 | 0.09 | 0.38 |
| Precentral gyrus | 0.94 ± 0.07 | 0.94 ± 0.03 | 0.97 | 0.99 |
| Straight gyrus | 1.00 ± 0.07 | 0.99 ± 0.05 | 0.71 | 0.92 |
| Anterior orbital gyrus | 0.93 ± 0.09 | 0.94 ± 0.06 | 0.69 | 0.92 |
| Lateral orbital gyrus | 0.91 ± 0.10 | 0.94 ± 0.06 | 0.43 | 0.69 |
| Medial orbital gyrus | 0.93 ± 0.07 | 0.93 ± 0.04 | 0.94 | 0.99 |
| Posterior orbital gyrus | 1.01 ± 0.08 | 0.99 ± 0.04 | 0.49 | 0.75 |
| Subcallosal area | 0.90 ± 0.12 | 0.89 ± 0.12 | 0.79 | 0.99 |
| Subgenual frontal cortex | 0.96 ± 0.09 | 0.93 ± 0.06 | 0.28 | 0.68 |
| Pre-subgenual frontal cortex | 1.06 ± 0.09 | 1.02 ± 0.09 | 0.28 | 0.68 |
| Cuneus | 1.08 ± 0.10 | 1.11 ± 0.06 | 0.42 | 0.69 |
| Lingual gyrus | 1.10 ± 0.09 | 1.09 ± 0.05 | 0.60 | 0.82 |
| Lateral remainder of occipital lobe | 0.91 ± 0.09 | 0.93 ± 0.04 | 0.41 | 0.69 |
| Hippocampus | 0.81 ± 0.06 | 0.85 ± 0.04 | 0.03 | 0.22 |
| Amygdala | 0.80 ± 0.08 | 0.82 ± 0.07 | 0.35 | 0.68 |
| Anterior temporal lobe lateral part | 0.75 ± 0.06 | 0.79 ± 0.06 | 0.12 | 0.44 |
| Anterior temporal lobe medial part | 0.74 ± 0.06 | 0.76 ± 0.03 | 0.34 | 0.68 |
| Parahippocampal and ambient gyri | 0.82 ± 0.07 | 0.81 ± 0.04 | 0.92 | 0.99 |
| Superior temporal gyrus anterior part | 0.80 ± 0.06 | 0.83 ± 0.07 | 0.32 | 0.68 |
| Superior temporal gyrus posterior part | 0.94 ± 0.08 | 1.00 ± 0.08 | 0.03* | 0.22 |
| Middle and inferior temporal gyrus | 0.82 ± 0.07 | 0.88 ± 0.04 | <0.01* | 0.09 |
| Fusiform gyrus | 0.78 ± 0.06 | 0.79 ± 0.04 | 0.58 | 0.82 |
| Posterior temporal lobe | 0.89 ± 0.06 | 0.93 ± 0.03 | 0.03* | 0.22 |
| Postcentral gyrus | 0.90 ± 0.07 | 0.90 ± 0.05 | 0.98 | 0.99 |
| Superior parietal gyrus | 0.90 ± 0.09 | 0.95 ± 0.05 | 0.07 | 0.35 |
| Inferiolateral remainder of parietal lobe | 0.86 ± 0.07 | 0.95 ± 0.05 | <0.01* | 0.03 * |
| Caudate nucleus | 0.64 ± 0.17 | 0.69 ± 0.07 | 0.35 | 0.68 |
| Nucleus accumbens | 1.00 ± 0.11 | 0.95 ± 0.09 | 0.18 | 0.61 |
| Putamen | 1.14 ± 0.08 | 1.12 ± 0.06 | 0.34 | 0.68 |
| Thalamus | 0.98 ± 0.10 | 1.01 ± 0.06 | 0.37 | 0.68 |
| Pallidum | 0.92 ± 0.09 | 0.92 ± 0.07 | 0.93 | 0.99 |
| Substantia nigra | 0.81 ± 0.08 | 0.84 ± 0.05 | 0.37 | 0.68 |
| Insula | 0.98 ± 0.07 | 0.98 ± 0.06 | 0.94 | 0.99 |
| Cingulate gyrus anterior part | 0.99 ± 0.10 | 0.99 ± 0.06 | 0.83 | 0.99 |
| Cingulate gyrus posterior part | 1.03 ± 0.08 | 1.11 ± 0.06 | <0.01* | 0.09 |
| Brainstem | 0.80 ± 0.04 | 0.82 ± 0.02 | 0.12 | 0.44 |
| Cerebellum | 1.00 ± 0.00 | 1.00 ± 0.00 | 0.51 | 0.75 |
| White matter | 0.72 ± 0.05 | 0.74 ± 0.03 | 0.28 | 0.68 |

* Statistically significant values.
