# Supplementary material for: Relative cerebral flow from dynamic PIB scans as an alternative for FDG scans in Alzheimer’s disease PET studies
Source: PLoS One. 2019 Jan 17;14(1):e0211000. doi: 10.1371/journal.pone.0211000 (PMC6336325; doi:10.1371/journal.pone.0211000)
Supplement: S9 Table — FDG SUVR values corrected for partial volume effects (expressed as mean ± standard deviation) for each region per subject group, and uncorrected and corrected for false discovery rate t-values from the t-test. (DOCX) [file pone.0211000.s013.docx]

| **Region** | **PIB+** | **PIB-** | **p-value^unc^** | **p-value^FDR^** |
| --- | --- | --- | --- | --- |
| Superior frontal gyrus | 1.47 ± 0.14 | 1.53 ± 0.10 | 0.17 | 0.48 |
| Middle frontal gyrus | 1.49 ± 0.13 | 1.60 ± 0.09 | 0.01* | 0.08 |
| Inferior frontal gyrus | 1.48 ± 0.16 | 1.53 ± 0.10 | 0.34 | 0.54 |
| Precentral gyrus | 1.70 ± 0.27 | 1.66 ± 0.12 | 0.58 | 0.62 |
| Straight gyrus | 1.29 ± 0.09 | 1.27 ± 0.09 | 0.59 | 0.62 |
| Anterior orbital gyrus | 1.41 ± 0.13 | 1.48 ± 0.10 | 0.11 | 0.38 |
| Lateral orbital gyrus | 1.41 ± 0.20 | 1.50 ± 0.14 | 0.19 | 0.48 |
| Medial orbital gyrus | 1.35 ± 0.14 | 1.40 ± 0.10 | 0.26 | 0.50 |
| Posterior orbital gyrus | 1.24 ± 0.12 | 1.27 ± 0.08 | 0.55 | 0.62 |
| Subcallosal area | 1.18 ± 0.19 | 1.17 ± 0.10 | 0.80 | 0.82 |
| Subgenual frontal cortex | 1.00 ± 0.08 | 0.91 ± 0.08 | < 0.01* | 0.03* |
| Pre-subgenual frontal cortex | 0.99 ± 0.17 | 0.95 ± 0.13 | 0.44 | 0.57 |
| Cuneus | 1.59 ± 0.21 | 1.64 ± 0.08 | 0.43 | 0.57 |
| Lingual gyrus | 1.45 ± 0.23 | 1.40 ± 0.09 | 0.51 | 0.60 |
| Lateral remainder of occipital lobe | 1.35 ± 0.15 | 1.40 ± 0.08 | 0.25 | 0.50 |
| Hippocampus | 0.73 ± 0.06 | 0.78 ± 0.07 | 0.03 | 0.15 |
| Amygdala | 0.77 ± 0.06 | 0.80 ± 0.06 | 0.10 | 0.38 |
| Anterior temporal lobe lateral part | 1.07 ± 0.12 | 1.11 ± 0.08 | 0.40 | 0.56 |
| Anterior temporal lobe medial part | 0.94 ± 0.06 | 0.92 ± 0.04 | 0.48 | 0.60 |
| Parahippocampal and ambient gyri | 0.95 ± 0.06 | 0.97 ± 0.07 | 0.41 | 0.56 |
| Superior temporal gyrus anterior part | 1.10 ± 0.11 | 1.08 ± 0.04 | 0.33 | 0.54 |
| Superior temporal gyrus posterior part | 1.36 ± 0.19 | 1.40 ± 0.10 | 0.58 | 0.62 |
| Middle and inferior temporal gyrus | 1.15 ± 0.13 | 1.25 ± 0.06 | 0.01* | 0.07 |
| Fusiform gyrus | 0.94 ± 0.06 | 0.92 ± 0.05 | 0.22 | 0.50 |
| Posterior temporal lobe | 1.11 ± 0.09 | 1.17 ± 0.06 | 0.03* | 0.15 |
| Postcentral gyrus | 1.67 ± 0.20 | 1.64 ± 0.08 | 0.51 | 0.60 |
| Superior parietal gyrus | 1.46 ± 0.12 | 1.61 ± 0.09 | < 0.01* | 0.01* |
| Inferiolateral remainder of parietal lobe | 1.31 ± 0.10 | 1.46 ± 0.08 | < 0.01* | < 0.01* |
| Caudate nucleus | 0.83 ± 0.28 | 0.93 ± 0.18 | 0.25 | 0.50 |
| Nucleus accumbens | 1.22 ± 0.15 | 1.29 ± 0.23 | 0.39 | 0.56 |
| Putamen | 1.25 ± 0.13 | 1.32 ± 0.12 | 0.17 | 0.47 |
| Thalamus | 0.98 ± 0.10 | 1.02 ± 0.10 | 0.24 | 0.50 |
| Pallidum | 0.93 ± 0.13 | 0.97 ± 0.10 | 0.35 | 0.54 |
| Substantia nigra | 0.82 ± 0.20 | 1.02 ± 0.18 | 0.01* | 0.06 |
| Insula | 0.99 ± 0.08 | 0.99 ± 0.07 | 0.94 | 0.94 |
| Cingulate gyrus anterior part | 1.21 ± 0.11 | 1.17 ± 0.07 | 0.28 | 0.50 |
| Cingulate gyrus posterior part | 1.36 ± 0.10 | 1.47 ± 0.10 | < 0.01* | 0.04* |
| Brainstem | 0.67 ± 0.04 | 0.69 ± 0.04 | 0.13 | 0.42 |
| Cerebellum | 1.00 ± 0.00 | 1.00 ± 0.00 | 0.31 | 0.54 |
| White matter | 0.37 ± 0.05 | 0.39 ± 0.03 | 0.10 | 0.38 |

* Statistically significant values.
