# Supplementary material for: Relative cerebral flow from dynamic PIB scans as an alternative for FDG scans in Alzheimer’s disease PET studies
Source: PLoS One. 2019 Jan 17;14(1):e0211000. doi: 10.1371/journal.pone.0211000 (PMC6336325; doi:10.1371/journal.pone.0211000)
Supplement: S11 Table — ePIB(20-130s) values corrected for partial volume effects (expressed as mean ± standard deviation) for each region per subject group, and uncorrected and corrected for false discovery rate t-values from the t-test. (DOCX) [file pone.0211000.s015.docx]

| **Region** | **PIB+** | **PIB-** | **p-value^unc^** | **p-value^FDR^** |
| --- | --- | --- | --- | --- |
| Superior frontal gyrus | 1.30 ± 0.10 | 1.27 ± 0.09 | 0.29 | 0.72 |
| Middle frontal gyrus | 1.27 ± 0.08 | 1.28 ± 0.10 | 0.75 | 0.90 |
| Inferior frontal gyrus | 1.33 ± 0.09 | 1.33 ± 0.10 | 0.96 | 0.99 |
| Precentral gyrus | 1.52 ± 0.18 | 1.46 ± 0.10 | 0.21 | 0.66 |
| Straight gyrus | 1.31 ± 0.10 | 1.22 ± 0.08 | 0.01 | 0.23 |
| Anterior orbital gyrus | 1.22 ± 0.13 | 1.20 ± 0.10 | 0.64 | 0.90 |
| Lateral orbital gyrus | 1.23 ± 0.19 | 1.25 ± 0.12 | 0.75 | 0.90 |
| Medial orbital gyrus | 1.23 ± 0.11 | 1.21 ± 0.10 | 0.65 | 0.90 |
| Posterior orbital gyrus | 1.28 ± 0.09 | 1.23 ± 0.09 | 0.16 | 0.59 |
| Subcallosal area | 1.47 ± 0.38 | 1.61 ± 0.72 | 0.51 | 0.82 |
| Subgenual frontal cortex | 1.20 ± 0.15 | 1.02 ± 0.11 | < 0.01 * | 0.05 * |
| Pre-subgenual frontal cortex | 1.12 ± 0.17 | 0.96 ± 0.19 | 0.02 * | 0.25 |
| Cuneus | 1.53 ± 0.18 | 1.51 ± 0.13 | 0.76 | 0.90 |
| Lingual gyrus | 1.42 ± 0.18 | 1.34 ± 0.11 | 0.16 | 0.59 |
| Lateral remainder of occipital lobe | 1.22 ± 0.16 | 1.22 ± 0.10 | 0.99 | 0.99 |
| Hippocampus | 0.72 ± 0.08 | 0.77 ± 0.08 | 0.08 | 0.59 |
| Amygdala | 0.87 ± 0.09 | 0.89 ± 0.13 | 0.69 | 0.90 |
| Anterior temporal lobe lateral part | 0.95 ± 0.09 | 0.95 ± 0.11 | 0.95 | 0.99 |
| Anterior temporal lobe medial part | 0.91 ± 0.06 | 0.90 ± 0.03 | 0.42 | 0.79 |
| Parahippocampal and ambient gyri | 1.05 ± 0.12 | 1.01 ± 0.09 | 0.33 | 0.73 |
| Superior temporal gyrus anterior part | 1.09 ± 0.13 | 1.09 ± 0.11 | 0.98 | 0.99 |
| Superior temporal gyrus posterior part | 1.34 ± 0.18 | 1.38 ± 0.18 | 0.54 | 0.84 |
| Middle and inferior temporal gyrus | 1.02 ± 0.10 | 1.07 ± 0.06 | 0.11 | 0.59 |
| Fusiform gyrus | 0.85 ± 0.09 | 0.83 ± 0.07 | 0.47 | 0.79 |
| Posterior temporal lobe | 1.05 ± 0.07 | 1.05 ± 0.06 | 0.81 | 0.92 |
| Postcentral gyrus | 1.51 ± 0.14 | 1.43 ± 0.13 | 0.14 | 0.59 |
| Superior parietal gyrus | 1.28 ± 0.12 | 1.31 ± 0.08 | 0.43 | 0.79 |
| Inferiolateral remainder of parietal lobe | 1.18 ± 0.10 | 1.23 ± 0.10 | 0.17 | 0.59 |
| Caudate nucleus | 0.67 ± 0.24 | 0.73 ± 0.11 | 0.41 | 0.79 |
| Nucleus accumbens | 1.27 ± 0.21 | 1.16 ± 0.24 | 0.18 | 0.59 |
| Putamen | 1.16 ± 0.09 | 1.15 ± 0.08 | 0.77 | 0.90 |
| Thalamus | 0.98 ± 0.13 | 1.01 ± 0.08 | 0.43 | 0.79 |
| Pallidum | 0.95 ± 0.14 | 0.95 ± 0.12 | 0.96 | 0.99 |
| Substantia nigra | 0.87 ± 0.18 | 0.91 ± 0.18 | 0.46 | 0.79 |
| Insula | 1.03 ± 0.08 | 1.00 ± 0.09 | 0.30 | 0.72 |
| Cingulate gyrus anterior part | 1.25 ± 0.12 | 1.15 ± 0.09 | 0.02 * | 0.23 |
| Cingulate gyrus posterior part | 1.29 ± 0.06 | 1.30 ± 0.07 | 0.72 | 0.90 |
| Brainstem | 0.76 ± 0.04 | 0.78 ± 0.04 | 0.15 | 0.59 |
| Cerebellum | 1.00 ± 0.00 | 1.00 ± 0.00 | 0.27 | 0.72 |
| White matter | 0.31 ± 0.03 | 0.33 ± 0.05 | 0.25 | 0.72 |

* Statistically significant values.
